# Supplementary material for: Experimental evidence demonstrating how freeze-thaw patterns affect spoilage of perishable cached food
Source: PLoS One. 2025 Apr 4;20(4):e0319043. doi: 10.1371/journal.pone.0319043 (PMC11970643; doi:10.1371/journal.pone.0319043)
Supplement: S1 Table — Historical climate data were obtained from Environment Canada at the Algonquin Provincial Park East Gate weather station (45°32’N, 78°54’W; https://weather.gc.ca/city/pages/on-29_metric_e.html). (PDF) [file pone.0319043.s001.pdf]

S1 Table. The number of freeze-thaw events in Oct., Nov. and Dec. from 2004 – 2019. Historical climate data were obtained from Environment Canada at the Algonquin Provincial Park East Gate weather station (45°32’N, 78°54’W; https://weather.gc.ca/city/pages/on-29_metric_e.html).

	Number of Freeze-thaw Events		
**Year**	**Oct.**	**Nov.**	**Dec.**
2004	11	22	10
2005	6	11	4
2006	8	13	15
2007	4	19	8
2008	12	14	6
2009	10	15	15
2010	11	19	5
2011	8	16	12
2012	4	19	9
2013	5	15	6
2014	5	11	10
2015	8	18	13
2016	7	13	14
2017	3	10	3
2018	10	8	11
2019	9	14	9
