# Supplementary material for: Experimental evidence demonstrating how freeze-thaw patterns affect spoilage of perishable cached food
Source: PLoS One. 2025 Apr 4;20(4):e0319043. doi: 10.1371/journal.pone.0319043 (PMC11970643; doi:10.1371/journal.pone.0319043)
Supplement: S2 Table — The number indicates the number of individual freeze-thaw events. Duration indicates the average duration (hrs) of a freeze or thaw in a given year. The average minimum and maximum temperatures provide the average lowest temperature reached in freezes and highest temperature in thaw events in a given year. (PDF) [file pone.0319043.s002.pdf]

S2 Table. The summary of freeze and thaw phases in the month of Nov. from Algonquin Provincial Park East Gate weather station (45°32'N, 78°54'W; 2004–2019). The number indicates the number of individual freeze-thaw events. Duration indicates the average duration (hrs) of a freeze or thaw in a given year. The average minimum and maximum temperatures provide the average lowest temperature reached in freezes and highest temperature in thaw events in a given year.

Year	No. of Nov. freeze-thaw events	Duration of ave. freeze phase (hrs)	Ave. min. temp. of freeze phase (°C)	Duration of ave. thaw phase (hrs)	Ave. max. temp. of thaw phases (°C)
2004	22	12.1	-6.4	20.3	3.7
2005	11	21.5	-6.3	27.0	5.0
2006	13	12.8	-4.6	42.5	6.5
2007	19	17.4	-8.5	20.5	3.3
2008	14	25.1	-6.9	26.1	5.4
2009	15	11.9	-5.7	35.4	8.3
2010	19	15.2	-6.7	22.7	4.9
2011	16	10.3	-5.2	33.8	6.3
2012	19	18.5	-6.5	19.4	4.3
2013	15	22.9	-8.3	25.1	3.6
2014	11	31.3	-6.9	32.4	3.2
2015	18	11.6	-4.4	28.4	5.1
2016	13	16.2	-4.1	39.3	9.4

2017	10	27.6	-8.6	42.9	5.7
2018	8	46.0	-9.8	44.0	2.1
2019	14	29.4	-8.6	28.3	2.0
**Mean**	15	20.6	-6.7	30.5	4.9
