# Supplementary material for: Experimental evidence demonstrating how freeze-thaw patterns affect spoilage of perishable cached food
Source: PLoS One. 2025 Apr 4;20(4):e0319043. doi: 10.1371/journal.pone.0319043 (PMC11970643; doi:10.1371/journal.pone.0319043)
Supplement: S3 Table — Each freezer was dedicated to simulating either the early freeze-thaw treatment, late-freeze-thaw treatment, low temperature control (held at −4.9 °C, no freeze-thaw events), or high temperature control (held at 1.1 °C, no freeze-thaw events). All simulations were 720 hours long and both treatments had eight freeze-thaw events each. (PDF) [file pone.0319043.s003.pdf]

S3 Table. Hourly programming schedule for the four freezers used in experiment 1 to test the predictions of the '*exacerbation hypothesis*'. Each freezer was dedicated to simulating either the early freeze-thaw treatment, late-freeze-thaw treatment, low temperature control (held at -4.9°C, no freeze-thaw events), or high temperature control (held at 1.1°C, no freeze-thaw events). All simulations were 720 hours long and both treatments had eight freeze-thaw events each.

Hour	Early freeze-thaw (°C)	Late freeze-thaw (°C)	Low temperature control (°C)	High temperature control (°C)
1	1.3	1.3	-4.9	1.1
2	1.3	1.3	-4.9	1.1
3	0.5	1.3	-4.9	1.1
4	-0.3	1.3	-4.9	1.1
5	-1.1	1.3	-4.9	1.1
6	-1.9	1.3	-4.9	1.1
7	-2.7	1.3	-4.9	1.1
8	-3.5	1.3	-4.9	1.1
9	-4.3	1.3	-4.9	1.1
10	-5.1	1.3	-4.9	1.1
11	-5.9	1.3	-4.9	1.1
12	-6.7	1.3	-4.9	1.1
13	-6.7	1.3	-4.9	1.1
14	-6.7	1.3	-4.9	1.1
15	-6.7	1.3	-4.9	1.1
16	-6.7	1.3	-4.9	1.1
17	-6.7	1.3	-4.9	1.1
18	-6.7	1.3	-4.9	1.1
19	-6.7	1.3	-4.9	1.1
20	-6.7	1.3	-4.9	1.1
21	-6.7	1.3	-4.9	1.1
22	-6.7	1.3	-4.9	1.1
23	-6.7	1.3	-4.9	1.1
24	-6.7	1.3	-4.9	1.1
25	-6.7	1.3	-4.9	1.1
26	-6.7	1.3	-4.9	1.1
27	-6.7	1.3	-4.9	1.1
28	-6.7	1.3	-4.9	1.1

29	-6.7	1.3	-4.9	1.1
30	-6.7	1.3	-4.9	1.1
31	-6.7	1.3	-4.9	1.1
32	-6.7	1.3	-4.9	1.1
33	-6.7	1.3	-4.9	1.1
34	-6.7	1.3	-4.9	1.1
35	-6.7	1.3	-4.9	1.1
36	-6.7	1.3	-4.9	1.1
37	-6.7	1.3	-4.9	1.1
38	-6.7	1.3	-4.9	1.1
39	-6.7	1.3	-4.9	1.1
40	-6.7	1.3	-4.9	1.1
41	-6.7	1.3	-4.9	1.1
42	-5.9	1.3	-4.9	1.1
43	-5.1	1.3	-4.9	1.1
44	-4.3	1.3	-4.9	1.1
45	-3.5	1.3	-4.9	1.1
46	-2.7	1.3	-4.9	1.1
47	-1.9	1.3	-4.9	1.1
48	-1.1	1.3	-4.9	1.1
49	-0.3	1.3	-4.9	1.1
50	0.5	1.3	-4.9	1.1
51	1.3	1.3	-4.9	1.1
52	2.1	1.3	-4.9	1.1
53	2.9	1.3	-4.9	1.1
54	3.7	1.3	-4.9	1.1
55	4.5	1.3	-4.9	1.1
56	4.5	1.3	-4.9	1.1
57	3.7	1.3	-4.9	1.1
58	2.9	1.3	-4.9	1.1
59	2.1	1.3	-4.9	1.1
60	1.3	1.3	-4.9	1.1
61	0.5	1.3	-4.9	1.1
62	-0.3	1.3	-4.9	1.1
63	-1.1	1.3	-4.9	1.1
64	-1.9	1.3	-4.9	1.1
65	-2.7	1.3	-4.9	1.1
66	-3.5	1.3	-4.9	1.1
67	-4.3	1.3	-4.9	1.1
68	-5.1	1.3	-4.9	1.1

69	-5.9	1.3	-4.9	1.1
70	-6.7	1.3	-4.9	1.1
71	-6.7	1.3	-4.9	1.1
72	-6.7	1.3	-4.9	1.1
73	-6.7	1.3	-4.9	1.1
74	-6.7	1.3	-4.9	1.1
75	-6.7	1.3	-4.9	1.1
76	-6.7	1.3	-4.9	1.1
77	-6.7	1.3	-4.9	1.1
78	-6.7	1.3	-4.9	1.1
79	-6.7	1.3	-4.9	1.1
80	-6.7	1.3	-4.9	1.1
81	-6.7	1.3	-4.9	1.1
82	-6.7	1.3	-4.9	1.1
83	-6.7	1.3	-4.9	1.1
84	-6.7	1.3	-4.9	1.1
85	-6.7	1.3	-4.9	1.1
86	-6.7	1.3	-4.9	1.1
87	-6.7	1.3	-4.9	1.1
88	-6.7	1.3	-4.9	1.1
89	-6.7	1.3	-4.9	1.1
90	-6.7	1.3	-4.9	1.1
91	-6.7	1.3	-4.9	1.1
92	-6.7	1.3	-4.9	1.1
93	-6.7	1.3	-4.9	1.1
94	-6.7	1.3	-4.9	1.1
95	-6.7	1.3	-4.9	1.1
96	-6.7	1.3	-4.9	1.1
97	-6.7	1.3	-4.9	1.1
98	-6.7	1.3	-4.9	1.1
99	-6.7	1.3	-4.9	1.1
100	-5.9	1.3	-4.9	1.1
101	-5.1	1.3	-4.9	1.1
102	-4.3	1.3	-4.9	1.1
103	-3.5	1.3	-4.9	1.1
104	-2.7	1.3	-4.9	1.1
105	-1.9	1.3	-4.9	1.1
106	-1.1	1.3	-4.9	1.1
107	-0.3	1.3	-4.9	1.1
108	0.5	1.3	-4.9	1.1

109	1.3	1.3	-4.9	1.1
110	2.1	1.3	-4.9	1.1
111	2.9	1.3	-4.9	1.1
112	3.7	1.3	-4.9	1.1
113	4.5	1.3	-4.9	1.1
114	4.5	1.3	-4.9	1.1
115	3.7	1.3	-4.9	1.1
116	2.9	1.3	-4.9	1.1
117	2.1	1.3	-4.9	1.1
118	1.3	1.3	-4.9	1.1
119	0.5	1.3	-4.9	1.1
120	-0.3	1.3	-4.9	1.1
121	-1.1	1.3	-4.9	1.1
122	-1.9	1.3	-4.9	1.1
123	-2.7	1.3	-4.9	1.1
124	-3.5	1.3	-4.9	1.1
125	-4.3	1.3	-4.9	1.1
126	-5.1	1.3	-4.9	1.1
127	-5.9	1.3	-4.9	1.1
128	-6.7	1.3	-4.9	1.1
129	-6.7	1.3	-4.9	1.1
130	-6.7	1.3	-4.9	1.1
131	-6.7	1.3	-4.9	1.1
132	-6.7	1.3	-4.9	1.1
133	-6.7	1.3	-4.9	1.1
134	-6.7	1.3	-4.9	1.1
135	-6.7	1.3	-4.9	1.1
136	-6.7	1.3	-4.9	1.1
137	-6.7	1.3	-4.9	1.1
138	-6.7	1.3	-4.9	1.1
139	-6.7	1.3	-4.9	1.1
140	-6.7	1.3	-4.9	1.1
141	-6.7	1.3	-4.9	1.1
142	-6.7	1.3	-4.9	1.1
143	-6.7	1.3	-4.9	1.1
144	-6.7	1.3	-4.9	1.1
145	-6.7	1.3	-4.9	1.1
146	-6.7	1.3	-4.9	1.1
147	-6.7	1.3	-4.9	1.1
148	-6.7	1.3	-4.9	1.1

149	-6.7	1.3	-4.9	1.1
150	-6.7	1.3	-4.9	1.1
151	-6.7	1.3	-4.9	1.1
152	-6.7	1.3	-4.9	1.1
153	-6.7	1.3	-4.9	1.1
154	-6.7	1.3	-4.9	1.1
155	-6.7	1.3	-4.9	1.1
156	-6.7	1.3	-4.9	1.1
157	-6.7	1.3	-4.9	1.1
158	-5.9	1.3	-4.9	1.1
159	-5.1	1.3	-4.9	1.1
160	-4.3	1.3	-4.9	1.1
161	-3.5	1.3	-4.9	1.1
162	-2.7	1.3	-4.9	1.1
163	-1.9	1.3	-4.9	1.1
164	-1.1	1.3	-4.9	1.1
165	-0.3	1.3	-4.9	1.1
166	0.5	1.3	-4.9	1.1
167	1.3	1.3	-4.9	1.1
168	2.1	1.3	-4.9	1.1
169	2.9	1.3	-4.9	1.1
170	3.7	1.3	-4.9	1.1
171	4.5	1.3	-4.9	1.1
172	4.5	1.3	-4.9	1.1
173	3.7	1.3	-4.9	1.1
174	2.9	1.3	-4.9	1.1
175	2.1	1.3	-4.9	1.1
176	1.3	1.3	-4.9	1.1
177	0.5	1.3	-4.9	1.1
178	-0.3	1.3	-4.9	1.1
179	-1.1	1.3	-4.9	1.1
180	-1.9	1.3	-4.9	1.1
181	-2.7	1.3	-4.9	1.1
182	-3.5	1.3	-4.9	1.1
183	-4.3	1.3	-4.9	1.1
184	-5.1	1.3	-4.9	1.1
185	-5.9	1.3	-4.9	1.1
186	-6.7	1.3	-4.9	1.1
187	-6.7	1.3	-4.9	1.1
188	-6.7	1.3	-4.9	1.1

189	-6.7	1.3	-4.9	1.1
190	-6.7	1.3	-4.9	1.1
191	-6.7	1.3	-4.9	1.1
192	-6.7	1.3	-4.9	1.1
193	-6.7	1.3	-4.9	1.1
194	-6.7	1.3	-4.9	1.1
195	-6.7	1.3	-4.9	1.1
196	-6.7	1.3	-4.9	1.1
197	-6.7	1.3	-4.9	1.1
198	-6.7	1.3	-4.9	1.1
199	-6.7	1.3	-4.9	1.1
200	-6.7	1.3	-4.9	1.1
201	-6.7	1.3	-4.9	1.1
202	-6.7	1.3	-4.9	1.1
203	-6.7	1.3	-4.9	1.1
204	-6.7	1.3	-4.9	1.1
205	-6.7	1.3	-4.9	1.1
206	-6.7	1.3	-4.9	1.1
207	-6.7	1.3	-4.9	1.1
208	-6.7	0.5	-4.9	1.1
209	-6.7	-0.3	-4.9	1.1
210	-6.7	-1.1	-4.9	1.1
211	-6.7	-1.9	-4.9	1.1
212	-6.7	-2.7	-4.9	1.1
213	-6.7	-3.5	-4.9	1.1
214	-6.7	-4.3	-4.9	1.1
215	-6.7	-5.1	-4.9	1.1
216	-5.9	-5.9	-4.9	1.1
217	-5.1	-6.7	-4.9	1.1
218	-4.3	-6.7	-4.9	1.1
219	-3.5	-6.7	-4.9	1.1
220	-2.7	-6.7	-4.9	1.1
221	-1.9	-6.7	-4.9	1.1
222	-1.1	-6.7	-4.9	1.1
223	-0.3	-6.7	-4.9	1.1
224	0.5	-6.7	-4.9	1.1
225	1.3	-6.7	-4.9	1.1
226	2.1	-6.7	-4.9	1.1
227	2.9	-6.7	-4.9	1.1
228	3.7	-6.7	-4.9	1.1

229	4.5	-6.7	-4.9	1.1
230	4.5	-6.7	-4.9	1.1
231	3.7	-6.7	-4.9	1.1
232	2.9	-6.7	-4.9	1.1
233	2.1	-6.7	-4.9	1.1
234	1.3	-6.7	-4.9	1.1
235	0.5	-6.7	-4.9	1.1
236	-0.3	-6.7	-4.9	1.1
237	-1.1	-6.7	-4.9	1.1
238	-1.9	-6.7	-4.9	1.1
239	-2.7	-6.7	-4.9	1.1
240	-3.5	-6.7	-4.9	1.1
241	-4.3	-6.7	-4.9	1.1
242	-5.1	-6.7	-4.9	1.1
243	-5.9	-6.7	-4.9	1.1
244	-6.7	-6.7	-4.9	1.1
245	-6.7	-6.7	-4.9	1.1
246	-6.7	-6.7	-4.9	1.1
247	-6.7	-5.9	-4.9	1.1
248	-6.7	-5.1	-4.9	1.1
249	-6.7	-4.3	-4.9	1.1
250	-6.7	-3.5	-4.9	1.1
251	-6.7	-2.7	-4.9	1.1
252	-6.7	-1.9	-4.9	1.1
253	-6.7	-1.1	-4.9	1.1
254	-6.7	-0.3	-4.9	1.1
255	-6.7	0.5	-4.9	1.1
256	-6.7	1.3	-4.9	1.1
257	-6.7	2.1	-4.9	1.1
258	-6.7	2.9	-4.9	1.1
259	-6.7	3.7	-4.9	1.1
260	-6.7	4.5	-4.9	1.1
261	-6.7	4.5	-4.9	1.1
262	-6.7	3.7	-4.9	1.1
263	-6.7	2.9	-4.9	1.1
264	-6.7	2.1	-4.9	1.1
265	-6.7	1.3	-4.9	1.1
266	-6.7	0.5	-4.9	1.1
267	-6.7	-0.3	-4.9	1.1
268	-6.7	-1.1	-4.9	1.1

269	-6.7	-1.9	-4.9	1.1
270	-6.7	-2.7	-4.9	1.1
271	-6.7	-3.5	-4.9	1.1
272	-6.7	-4.3	-4.9	1.1
273	-6.7	-5.1	-4.9	1.1
274	-5.9	-5.9	-4.9	1.1
275	-5.1	-6.7	-4.9	1.1
276	-4.3	-6.7	-4.9	1.1
277	-3.5	-6.7	-4.9	1.1
278	-2.7	-6.7	-4.9	1.1
279	-1.9	-6.7	-4.9	1.1
280	-1.1	-6.7	-4.9	1.1
281	-0.3	-6.7	-4.9	1.1
282	0.5	-6.7	-4.9	1.1
283	1.3	-6.7	-4.9	1.1
284	2.1	-6.7	-4.9	1.1
285	2.9	-6.7	-4.9	1.1
286	3.7	-6.7	-4.9	1.1
287	4.5	-6.7	-4.9	1.1
288	4.5	-6.7	-4.9	1.1
289	3.7	-6.7	-4.9	1.1
290	2.9	-6.7	-4.9	1.1
291	2.1	-6.7	-4.9	1.1
292	1.3	-6.7	-4.9	1.1
293	0.5	-6.7	-4.9	1.1
294	-0.3	-6.7	-4.9	1.1
295	-1.1	-6.7	-4.9	1.1
296	-1.9	-6.7	-4.9	1.1
297	-2.7	-6.7	-4.9	1.1
298	-3.5	-6.7	-4.9	1.1
299	-4.3	-6.7	-4.9	1.1
300	-5.1	-6.7	-4.9	1.1
301	-5.9	-6.7	-4.9	1.1
302	-6.7	-6.7	-4.9	1.1
303	-6.7	-6.7	-4.9	1.1
304	-6.7	-6.7	-4.9	1.1
305	-6.7	-5.9	-4.9	1.1
306	-6.7	-5.1	-4.9	1.1
307	-6.7	-4.3	-4.9	1.1
308	-6.7	-3.5	-4.9	1.1

309	-6.7	-2.7	-4.9	1.1
310	-6.7	-1.9	-4.9	1.1
311	-6.7	-1.1	-4.9	1.1
312	-6.7	-0.3	-4.9	1.1
313	-6.7	0.5	-4.9	1.1
314	-6.7	1.3	-4.9	1.1
315	-6.7	2.1	-4.9	1.1
316	-6.7	2.9	-4.9	1.1
317	-6.7	3.7	-4.9	1.1
318	-6.7	4.5	-4.9	1.1
319	-6.7	4.5	-4.9	1.1
320	-6.7	3.7	-4.9	1.1
321	-6.7	2.9	-4.9	1.1
322	-6.7	2.1	-4.9	1.1
323	-6.7	1.3	-4.9	1.1
324	-6.7	0.5	-4.9	1.1
325	-6.7	-0.3	-4.9	1.1
326	-6.7	-1.1	-4.9	1.1
327	-6.7	-1.9	-4.9	1.1
328	-6.7	-2.7	-4.9	1.1
329	-6.7	-3.5	-4.9	1.1
330	-6.7	-4.3	-4.9	1.1
331	-6.7	-5.1	-4.9	1.1
332	-5.9	-5.9	-4.9	1.1
333	-5.1	-6.7	-4.9	1.1
334	-4.3	-6.7	-4.9	1.1
335	-3.5	-6.7	-4.9	1.1
336	-2.7	-6.7	-4.9	1.1
337	-1.9	-6.7	-4.9	1.1
338	-1.1	-6.7	-4.9	1.1
339	-0.3	-6.7	-4.9	1.1
340	0.5	-6.7	-4.9	1.1
341	1.3	-6.7	-4.9	1.1
342	2.1	-6.7	-4.9	1.1
343	2.9	-6.7	-4.9	1.1
344	3.7	-6.7	-4.9	1.1
345	4.5	-6.7	-4.9	1.1
346	4.5	-6.7	-4.9	1.1
347	3.7	-6.7	-4.9	1.1
348	2.9	-6.7	-4.9	1.1

349	2.1	-6.7	-4.9	1.1
350	1.3	-6.7	-4.9	1.1
351	0.5	-6.7	-4.9	1.1
352	-0.3	-6.7	-4.9	1.1
353	-1.1	-6.7	-4.9	1.1
354	-1.9	-6.7	-4.9	1.1
355	-2.7	-6.7	-4.9	1.1
356	-3.5	-6.7	-4.9	1.1
357	-4.3	-6.7	-4.9	1.1
358	-5.1	-6.7	-4.9	1.1
359	-5.9	-6.7	-4.9	1.1
360	-6.7	-6.7	-4.9	1.1
361	-6.7	-6.7	-4.9	1.1
362	-6.7	-6.7	-4.9	1.1
363	-6.7	-5.9	-4.9	1.1
364	-6.7	-5.1	-4.9	1.1
365	-6.7	-4.3	-4.9	1.1
366	-6.7	-3.5	-4.9	1.1
367	-6.7	-2.7	-4.9	1.1
368	-6.7	-1.9	-4.9	1.1
369	-6.7	-1.1	-4.9	1.1
370	-6.7	-0.3	-4.9	1.1
371	-6.7	0.5	-4.9	1.1
372	-6.7	1.3	-4.9	1.1
373	-6.7	2.1	-4.9	1.1
374	-6.7	2.9	-4.9	1.1
375	-6.7	3.7	-4.9	1.1
376	-6.7	4.5	-4.9	1.1
377	-6.7	4.5	-4.9	1.1
378	-6.7	3.7	-4.9	1.1
379	-6.7	2.9	-4.9	1.1
380	-6.7	2.1	-4.9	1.1
381	-6.7	1.3	-4.9	1.1
382	-6.7	0.5	-4.9	1.1
383	-6.7	-0.3	-4.9	1.1
384	-6.7	-1.1	-4.9	1.1
385	-6.7	-1.9	-4.9	1.1
386	-6.7	-2.7	-4.9	1.1
387	-6.7	-3.5	-4.9	1.1
388	-6.7	-4.3	-4.9	1.1

389	-6.7	-5.1	-4.9	1.1
390	-5.9	-5.9	-4.9	1.1
391	-5.1	-6.7	-4.9	1.1
392	-4.3	-6.7	-4.9	1.1
393	-3.5	-6.7	-4.9	1.1
394	-2.7	-6.7	-4.9	1.1
395	-1.9	-6.7	-4.9	1.1
396	-1.1	-6.7	-4.9	1.1
397	-0.3	-6.7	-4.9	1.1
398	0.5	-6.7	-4.9	1.1
399	1.3	-6.7	-4.9	1.1
400	2.1	-6.7	-4.9	1.1
401	2.9	-6.7	-4.9	1.1
402	3.7	-6.7	-4.9	1.1
403	4.5	-6.7	-4.9	1.1
404	4.5	-6.7	-4.9	1.1
405	3.7	-6.7	-4.9	1.1
406	2.9	-6.7	-4.9	1.1
407	2.1	-6.7	-4.9	1.1
408	1.3	-6.7	-4.9	1.1
409	0.5	-6.7	-4.9	1.1
410	-0.3	-6.7	-4.9	1.1
411	-1.1	-6.7	-4.9	1.1
412	-1.9	-6.7	-4.9	1.1
413	-2.7	-6.7	-4.9	1.1
414	-3.5	-6.7	-4.9	1.1
415	-4.3	-6.7	-4.9	1.1
416	-5.1	-6.7	-4.9	1.1
417	-5.9	-6.7	-4.9	1.1
418	-6.7	-6.7	-4.9	1.1
419	-6.7	-6.7	-4.9	1.1
420	-6.7	-6.7	-4.9	1.1
421	-6.7	-5.9	-4.9	1.1
422	-6.7	-5.1	-4.9	1.1
423	-6.7	-4.3	-4.9	1.1
424	-6.7	-3.5	-4.9	1.1
425	-6.7	-2.7	-4.9	1.1
426	-6.7	-1.9	-4.9	1.1
427	-6.7	-1.1	-4.9	1.1
428	-6.7	-0.3	-4.9	1.1

429	-6.7	0.5	-4.9	1.1
430	-6.7	1.3	-4.9	1.1
431	-6.7	2.1	-4.9	1.1
432	-6.7	2.9	-4.9	1.1
433	-6.7	3.7	-4.9	1.1
434	-6.7	4.5	-4.9	1.1
435	-6.7	4.5	-4.9	1.1
436	-6.7	3.7	-4.9	1.1
437	-6.7	2.9	-4.9	1.1
438	-6.7	2.1	-4.9	1.1
439	-6.7	1.3	-4.9	1.1
440	-6.7	0.5	-4.9	1.1
441	-6.7	-0.3	-4.9	1.1
442	-6.7	-1.1	-4.9	1.1
443	-6.7	-1.9	-4.9	1.1
444	-6.7	-2.7	-4.9	1.1
445	-6.7	-3.5	-4.9	1.1
446	-6.7	-4.3	-4.9	1.1
447	-6.7	-5.1	-4.9	1.1
448	-5.9	-5.9	-4.9	1.1
449	-5.1	-6.7	-4.9	1.1
450	-4.3	-6.7	-4.9	1.1
451	-3.5	-6.7	-4.9	1.1
452	-2.7	-6.7	-4.9	1.1
453	-1.9	-6.7	-4.9	1.1
454	-1.1	-6.7	-4.9	1.1
455	-0.3	-6.7	-4.9	1.1
456	0.5	-6.7	-4.9	1.1
457	1.3	-6.7	-4.9	1.1
458	2.1	-6.7	-4.9	1.1
459	2.9	-6.7	-4.9	1.1
460	3.7	-6.7	-4.9	1.1
461	4.5	-6.7	-4.9	1.1
462	4.5	-6.7	-4.9	1.1
463	3.7	-6.7	-4.9	1.1
464	2.9	-6.7	-4.9	1.1
465	2.1	-6.7	-4.9	1.1
466	1.3	-6.7	-4.9	1.1
467	0.5	-6.7	-4.9	1.1
468	-0.3	-6.7	-4.9	1.1

469	-1.1	-6.7	-4.9	1.1
470	-1.9	-6.7	-4.9	1.1
471	-2.7	-6.7	-4.9	1.1
472	-3.5	-6.7	-4.9	1.1
473	-4.3	-6.7	-4.9	1.1
474	-5.1	-6.7	-4.9	1.1
475	-5.9	-6.7	-4.9	1.1
476	-6.7	-6.7	-4.9	1.1
477	-6.7	-6.7	-4.9	1.1
478	-6.7	-6.7	-4.9	1.1
479	-6.7	-5.9	-4.9	1.1
480	-6.7	-5.1	-4.9	1.1
481	-6.7	-4.3	-4.9	1.1
482	-6.7	-3.5	-4.9	1.1
483	-6.7	-2.7	-4.9	1.1
484	-6.7	-1.9	-4.9	1.1
485	-6.7	-1.1	-4.9	1.1
486	-6.7	-0.3	-4.9	1.1
487	-6.7	0.5	-4.9	1.1
488	-6.7	1.3	-4.9	1.1
489	-6.7	2.1	-4.9	1.1
490	-6.7	2.9	-4.9	1.1
491	-6.7	3.7	-4.9	1.1
492	-6.7	4.5	-4.9	1.1
493	-6.7	4.5	-4.9	1.1
494	-6.7	3.7	-4.9	1.1
495	-6.7	2.9	-4.9	1.1
496	-6.7	2.1	-4.9	1.1
497	-6.7	1.3	-4.9	1.1
498	-6.7	0.5	-4.9	1.1
499	-6.7	-0.3	-4.9	1.1
500	-6.7	-1.1	-4.9	1.1
501	-6.7	-1.9	-4.9	1.1
502	-6.7	-2.7	-4.9	1.1
503	-6.7	-3.5	-4.9	1.1
504	-6.7	-4.3	-4.9	1.1
505	-6.7	-5.1	-4.9	1.1
506	-5.9	-5.9	-4.9	1.1
507	-5.1	-6.7	-4.9	1.1
508	-4.3	-6.7	-4.9	1.1

509	-3.5	-6.7	-4.9	1.1
510	-2.7	-6.7	-4.9	1.1
511	-1.9	-6.7	-4.9	1.1
512	-1.1	-6.7	-4.9	1.1
513	-0.3	-6.7	-4.9	1.1
514	0.5	-6.7	-4.9	1.1
515	1.3	-6.7	-4.9	1.1
516	1.3	-6.7	-4.9	1.1
517	1.3	-6.7	-4.9	1.1
518	1.3	-6.7	-4.9	1.1
519	1.3	-6.7	-4.9	1.1
520	1.3	-6.7	-4.9	1.1
521	1.3	-6.7	-4.9	1.1
522	1.3	-6.7	-4.9	1.1
523	1.3	-6.7	-4.9	1.1
524	1.3	-6.7	-4.9	1.1
525	1.3	-6.7	-4.9	1.1
526	1.3	-6.7	-4.9	1.1
527	1.3	-6.7	-4.9	1.1
528	1.3	-6.7	-4.9	1.1
529	1.3	-6.7	-4.9	1.1
530	1.3	-6.7	-4.9	1.1
531	1.3	-6.7	-4.9	1.1
532	1.3	-6.7	-4.9	1.1
533	1.3	-6.7	-4.9	1.1
534	1.3	-6.7	-4.9	1.1
535	1.3	-6.7	-4.9	1.1
536	1.3	-6.7	-4.9	1.1
537	1.3	-5.9	-4.9	1.1
538	1.3	-5.1	-4.9	1.1
539	1.3	-4.3	-4.9	1.1
540	1.3	-3.5	-4.9	1.1
541	1.3	-2.7	-4.9	1.1
542	1.3	-1.9	-4.9	1.1
543	1.3	-1.1	-4.9	1.1
544	1.3	-0.3	-4.9	1.1
545	1.3	0.5	-4.9	1.1
546	1.3	1.3	-4.9	1.1
547	1.3	2.1	-4.9	1.1
548	1.3	2.9	-4.9	1.1

549	1.3	3.7	-4.9	1.1
550	1.3	4.5	-4.9	1.1
551	1.3	4.5	-4.9	1.1
552	1.3	3.7	-4.9	1.1
553	1.3	2.9	-4.9	1.1
554	1.3	2.1	-4.9	1.1
555	1.3	1.3	-4.9	1.1
556	1.3	0.5	-4.9	1.1
557	1.3	-0.3	-4.9	1.1
558	1.3	-1.1	-4.9	1.1
559	1.3	-1.9	-4.9	1.1
560	1.3	-2.7	-4.9	1.1
561	1.3	-3.5	-4.9	1.1
562	1.3	-4.3	-4.9	1.1
563	1.3	-5.1	-4.9	1.1
564	1.3	-5.9	-4.9	1.1
565	1.3	-6.7	-4.9	1.1
566	1.3	-6.7	-4.9	1.1
567	1.3	-6.7	-4.9	1.1
568	1.3	-6.7	-4.9	1.1
569	1.3	-6.7	-4.9	1.1
570	1.3	-6.7	-4.9	1.1
571	1.3	-6.7	-4.9	1.1
572	1.3	-6.7	-4.9	1.1
573	1.3	-6.7	-4.9	1.1
574	1.3	-6.7	-4.9	1.1
575	1.3	-6.7	-4.9	1.1
576	1.3	-6.7	-4.9	1.1
577	1.3	-6.7	-4.9	1.1
578	1.3	-6.7	-4.9	1.1
579	1.3	-6.7	-4.9	1.1
580	1.3	-6.7	-4.9	1.1
581	1.3	-6.7	-4.9	1.1
582	1.3	-6.7	-4.9	1.1
583	1.3	-6.7	-4.9	1.1
584	1.3	-6.7	-4.9	1.1
585	1.3	-6.7	-4.9	1.1
586	1.3	-6.7	-4.9	1.1
587	1.3	-6.7	-4.9	1.1
588	1.3	-6.7	-4.9	1.1

589	1.3	-6.7	-4.9	1.1
590	1.3	-6.7	-4.9	1.1
591	1.3	-6.7	-4.9	1.1
592	1.3	-6.7	-4.9	1.1
593	1.3	-6.7	-4.9	1.1
594	1.3	-6.7	-4.9	1.1
595	1.3	-5.9	-4.9	1.1
596	1.3	-5.1	-4.9	1.1
597	1.3	-4.3	-4.9	1.1
598	1.3	-3.5	-4.9	1.1
599	1.3	-2.7	-4.9	1.1
600	1.3	-1.9	-4.9	1.1
601	1.3	-1.1	-4.9	1.1
602	1.3	-0.3	-4.9	1.1
603	1.3	0.5	-4.9	1.1
604	1.3	1.3	-4.9	1.1
605	1.3	2.1	-4.9	1.1
606	1.3	2.9	-4.9	1.1
607	1.3	3.7	-4.9	1.1
608	1.3	4.5	-4.9	1.1
609	1.3	4.5	-4.9	1.1
610	1.3	3.7	-4.9	1.1
611	1.3	2.9	-4.9	1.1
612	1.3	2.1	-4.9	1.1
613	1.3	1.3	-4.9	1.1
614	1.3	0.5	-4.9	1.1
615	1.3	-0.3	-4.9	1.1
616	1.3	-1.1	-4.9	1.1
617	1.3	-1.9	-4.9	1.1
618	1.3	-2.7	-4.9	1.1
619	1.3	-3.5	-4.9	1.1
620	1.3	-4.3	-4.9	1.1
621	1.3	-5.1	-4.9	1.1
622	1.3	-5.9	-4.9	1.1
623	1.3	-6.7	-4.9	1.1
624	1.3	-6.7	-4.9	1.1
625	1.3	-6.7	-4.9	1.1
626	1.3	-6.7	-4.9	1.1
627	1.3	-6.7	-4.9	1.1
628	1.3	-6.7	-4.9	1.1

629	1.3	-6.7	-4.9	1.1
630	1.3	-6.7	-4.9	1.1
631	1.3	-6.7	-4.9	1.1
632	1.3	-6.7	-4.9	1.1
633	1.3	-6.7	-4.9	1.1
634	1.3	-6.7	-4.9	1.1
635	1.3	-6.7	-4.9	1.1
636	1.3	-6.7	-4.9	1.1
637	1.3	-6.7	-4.9	1.1
638	1.3	-6.7	-4.9	1.1
639	1.3	-6.7	-4.9	1.1
640	1.3	-6.7	-4.9	1.1
641	1.3	-6.7	-4.9	1.1
642	1.3	-6.7	-4.9	1.1
643	1.3	-6.7	-4.9	1.1
644	1.3	-6.7	-4.9	1.1
645	1.3	-6.7	-4.9	1.1
646	1.3	-6.7	-4.9	1.1
647	1.3	-6.7	-4.9	1.1
648	1.3	-6.7	-4.9	1.1
649	1.3	-6.7	-4.9	1.1
650	1.3	-6.7	-4.9	1.1
651	1.3	-6.7	-4.9	1.1
652	1.3	-6.7	-4.9	1.1
653	1.3	-5.9	-4.9	1.1
654	1.3	-5.1	-4.9	1.1
655	1.3	-4.3	-4.9	1.1
656	1.3	-3.5	-4.9	1.1
657	1.3	-2.7	-4.9	1.1
658	1.3	-1.9	-4.9	1.1
659	1.3	-1.1	-4.9	1.1
660	1.3	-0.3	-4.9	1.1
661	1.3	0.5	-4.9	1.1
662	1.3	1.3	-4.9	1.1
663	1.3	2.1	-4.9	1.1
664	1.3	2.9	-4.9	1.1
665	1.3	3.7	-4.9	1.1
666	1.3	4.5	-4.9	1.1
667	1.3	4.5	-4.9	1.1
668	1.3	3.7	-4.9	1.1

669	1.3	2.9	-4.9	1.1
670	1.3	2.1	-4.9	1.1
671	1.3	1.3	-4.9	1.1
672	1.3	0.5	-4.9	1.1
673	1.3	-0.3	-4.9	1.1
674	1.3	-1.1	-4.9	1.1
675	1.3	-1.9	-4.9	1.1
676	1.3	-2.7	-4.9	1.1
677	1.3	-3.5	-4.9	1.1
678	1.3	-4.3	-4.9	1.1
679	1.3	-5.1	-4.9	1.1
680	1.3	-5.9	-4.9	1.1
681	1.3	-6.7	-4.9	1.1
682	1.3	-6.7	-4.9	1.1
683	1.3	-6.7	-4.9	1.1
684	1.3	-6.7	-4.9	1.1
685	1.3	-6.7	-4.9	1.1
686	1.3	-6.7	-4.9	1.1
687	1.3	-6.7	-4.9	1.1
688	1.3	-6.7	-4.9	1.1
689	1.3	-6.7	-4.9	1.1
690	1.3	-6.7	-4.9	1.1
691	1.3	-6.7	-4.9	1.1
692	1.3	-6.7	-4.9	1.1
693	1.3	-6.7	-4.9	1.1
694	1.3	-6.7	-4.9	1.1
695	1.3	-6.7	-4.9	1.1
696	1.3	-6.7	-4.9	1.1
697	1.3	-6.7	-4.9	1.1
698	1.3	-6.7	-4.9	1.1
699	1.3	-6.7	-4.9	1.1
700	1.3	-6.7	-4.9	1.1
701	1.3	-6.7	-4.9	1.1
702	1.3	-6.7	-4.9	1.1
703	1.3	-6.7	-4.9	1.1
704	1.3	-6.7	-4.9	1.1
705	1.3	-6.7	-4.9	1.1
706	1.3	-6.7	-4.9	1.1
707	1.3	-6.7	-4.9	1.1
708	1.3	-6.7	-4.9	1.1

709	1.3	-6.7	-4.9	1.1
710	1.3	-6.7	-4.9	1.1
711	1.3	-5.9	-4.9	1.1
712	1.3	-5.1	-4.9	1.1
713	1.3	-4.3	-4.9	1.1
714	1.3	-3.5	-4.9	1.1
715	1.3	-2.7	-4.9	1.1
716	1.3	-1.9	-4.9	1.1
717	1.3	-1.1	-4.9	1.1
718	1.3	-0.3	-4.9	1.1
719	1.3	0.5	-4.9	1.1
720	1.3	1.3	-4.9	1.1
