# Supplementary material for: Experimental evidence demonstrating how freeze-thaw patterns affect spoilage of perishable cached food
Source: PLoS One. 2025 Apr 4;20(4):e0319043. doi: 10.1371/journal.pone.0319043 (PMC11970643; doi:10.1371/journal.pone.0319043)
Supplement: S4 Table — Each freezer was dedicated to simulating either the low frequency freeze-thaw treatment (8 freeze-thaw events), medium frequency freeze-thaw treatment (15 freeze-thaw events), high frequency freeze-thaw treatment (22 freeze-thaw events), or the control (held at −1.6 °C, no freeze-thaw events). All simulations were 720 hours long and all treatment groups had an identical total duration below freezing (368 hrs) and above-freezing (352 hrs) post-freeze. (PDF) [file pone.0319043.s004.pdf]

S4 Table. Hourly programming schedule for all freezers used in experiment 2 to test the predictions of the '*frequency hypothesis'* and the '*continuous thaw hypothesis*'. Each freezer was dedicated to simulating either the low frequency freeze-thaw treatment (8 freeze-thaw events), medium frequency freeze-thaw treatment (15 freeze-thaw events), high frequency freeze-thaw treatment (22 freeze-thaw events), or the control (held at -1.6°C, no freeze-thaw events). All simulations were 720 hours long and all treatment groups had an identical total duration below freezing (368 hrs) and above-freezing (352 hrs) post-freeze.

Hour	Low freq. freeze-thaw (°C)	Med freq. freeze-thaw (°C)	High freq. freeze-thaw (°C)	Control (°C)
1	-1.9	-1.9	-1.9	-1.6
2	-2.7	-2.7	-2.7	-1.6
3	-3.5	-3.5	-3.5	-1.6
4	-4.3	-4.3	-4.3	-1.6
5	-5.1	-5.1	-5.1	-1.6
6	-5.9	-5.9	-5.9	-1.6
7	-6.7	-6.7	-6.7	-1.6
8	-7.5	-7.5	-7.5	-1.6
9	-7.5	-7.5	-7.5	-1.6
10	-7.5	-7.5	-7.5	-1.6
11	-7.5	-7.5	-6.7	-1.6
12	-7.5	-7.5	-5.9	-1.6
13	-7.5	-7.5	-5.1	-1.6
14	-7.5	-7.5	-4.3	-1.6
15	-7.5	-7.5	-3.5	-1.6
16	-7.5	-7.5	-2.7	-1.6
17	-7.5	-7.5	-1.9	-1.6
18	-7.5	-6.7	-1.1	-1.6
19	-7.5	-5.9	-0.3	-1.6
20	-7.5	-5.1	0.5	-1.6
21	-7.5	-4.3	1.3	-1.6
22	-7.5	-3.5	2.1	-1.6
23	-7.5	-2.7	2.9	-1.6
24	-7.5	-1.9	3.7	-1.6
25	-7.5	-1.1	4.5	-1.6

26	-7.5	-0.3	4.5	-1.6
27	-7.5	0.5	3.7	-1.6
28	-7.5	1.3	2.9	-1.6
29	-7.5	2.1	2.1	-1.6
30	-7.5	2.9	1.3	-1.6
31	-7.5	3.7	0.5	-1.6
32	-7.5	4.5	-0.3	-1.6
33	-7.5	4.5	-1.1	-1.6
34	-7.5	4.5	-1.9	-1.6
35	-7.5	4.5	-2.7	-1.6
36	-7.5	4.5	-3.5	-1.6
37	-7.5	4.5	-4.3	-1.6
38	-7.5	4.5	-5.1	-1.6
39	-6.7	4.5	-5.9	-1.6
40	-5.9	4.5	-6.7	-1.6
41	-5.1	4.5	-7.5	-1.6
42	-4.3	3.7	-7.5	-1.6
43	-3.5	2.9	-7.5	-1.6
44	-2.7	2.1	-6.7	-1.6
45	-1.9	1.3	-5.9	-1.6
46	-1.1	0.5	-5.1	-1.6
47	-0.3	-0.3	-4.3	-1.6
48	0.5	-1.1	-3.5	-1.6
49	1.3	-1.9	-2.7	-1.6
50	2.1	-2.7	-1.9	-1.6
51	2.9	-3.5	-1.1	-1.6
52	3.7	-4.3	-0.3	-1.6
53	4.5	-5.1	0.5	-1.6
54	4.5	-5.9	1.3	-1.6
55	4.5	-6.7	2.1	-1.6
56	4.5	-7.5	2.9	-1.6
57	4.5	-7.5	3.7	-1.6
58	4.5	-7.5	4.5	-1.6
59	4.5	-7.5	4.5	-1.6
60	4.5	-7.5	3.7	-1.6
61	4.5	-7.5	2.9	-1.6
62	4.5	-7.5	2.1	-1.6
63	4.5	-7.5	1.3	-1.6
64	4.5	-7.5	0.5	-1.6
65	4.5	-7.5	-0.3	-1.6

66	4.5	-7.5	-1.1	-1.6
67	4.5	-6.7	-1.9	-1.6
68	4.5	-5.9	-2.7	-1.6
69	4.5	-5.1	-3.5	-1.6
70	4.5	-4.3	-4.3	-1.6
71	4.5	-3.5	-5.1	-1.6
72	4.5	-2.7	-5.9	-1.6
73	4.5	-1.9	-6.7	-1.6
74	4.5	-1.1	-7.5	-1.6
75	4.5	-0.3	-7.5	-1.6
76	4.5	0.5	-6.7	-1.6
77	4.5	1.3	-5.9	-1.6
78	4.5	2.1	-5.1	-1.6
79	4.5	2.9	-4.3	-1.6
80	4.5	3.7	-3.5	-1.6
81	4.5	4.5	-2.7	-1.6
82	4.5	4.5	-1.9	-1.6
83	3.7	4.5	-1.1	-1.6
84	2.9	4.5	-0.3	-1.6
85	2.1	4.5	0.5	-1.6
86	1.3	4.5	1.3	-1.6
87	0.5	4.5	2.1	-1.6
88	-0.3	4.5	2.9	-1.6
89	-1.1	4.5	3.7	-1.6
90	-1.9	3.7	4.5	-1.6
91	-2.7	2.9	4.5	-1.6
92	-3.5	2.1	3.7	-1.6
93	-4.3	1.3	2.9	-1.6
94	-5.1	0.5	2.1	-1.6
95	-5.9	-0.3	1.3	-1.6
96	-6.7	-1.1	0.5	-1.6
97	-7.5	-1.9	-0.3	-1.6
98	-7.5	-2.7	-1.1	-1.6
99	-7.5	-3.5	-1.9	-1.6
100	-7.5	-4.3	-2.7	-1.6
101	-7.5	-5.1	-3.5	-1.6
102	-7.5	-5.9	-4.3	-1.6
103	-7.5	-6.7	-5.1	-1.6
104	-7.5	-7.5	-5.9	-1.6
105	-7.5	-7.5	-6.7	-1.6

106	-7.5	-7.5	-7.5	-1.6
107	-7.5	-7.5	-7.5	-1.6
108	-7.5	-7.5	-7.5	-1.6
109	-7.5	-7.5	-6.7	-1.6
110	-7.5	-7.5	-5.9	-1.6
111	-7.5	-7.5	-5.1	-1.6
112	-7.5	-7.5	-4.3	-1.6
113	-7.5	-7.5	-3.5	-1.6
114	-7.5	-6.7	-2.7	-1.6
115	-7.5	-5.9	-1.9	-1.6
116	-7.5	-5.1	-1.1	-1.6
117	-7.5	-4.3	-0.3	-1.6
118	-7.5	-3.5	0.5	-1.6
119	-7.5	-2.7	1.3	-1.6
120	-7.5	-1.9	2.1	-1.6
121	-7.5	-1.1	2.9	-1.6
122	-7.5	-0.3	3.7	-1.6
123	-7.5	0.5	4.5	-1.6
124	-7.5	1.3	4.5	-1.6
125	-7.5	2.1	3.7	-1.6
126	-7.5	2.9	2.9	-1.6
127	-7.5	3.7	2.1	-1.6
128	-7.5	4.5	1.3	-1.6
129	-6.7	4.5	0.5	-1.6
130	-5.9	4.5	-0.3	-1.6
131	-5.1	4.5	-1.1	-1.6
132	-4.3	4.5	-1.9	-1.6
133	-3.5	4.5	-2.7	-1.6
134	-2.7	4.5	-3.5	-1.6
135	-1.9	4.5	-4.3	-1.6
136	-1.1	4.5	-5.1	-1.6
137	-0.3	4.5	-5.9	-1.6
138	0.5	3.7	-6.7	-1.6
139	1.3	2.9	-7.5	-1.6
140	2.1	2.1	-7.5	-1.6
141	2.9	1.3	-7.5	-1.6
142	3.7	0.5	-6.7	-1.6
143	4.5	-0.3	-5.9	-1.6
144	4.5	-1.1	-5.1	-1.6
145	4.5	-1.9	-4.3	-1.6

146	4.5	-2.7	-3.5	-1.6
147	4.5	-3.5	-2.7	-1.6
148	4.5	-4.3	-1.9	-1.6
149	4.5	-5.1	-1.1	-1.6
150	4.5	-5.9	-0.3	-1.6
151	4.5	-6.7	0.5	-1.6
152	4.5	-7.5	1.3	-1.6
153	4.5	-7.5	2.1	-1.6
154	4.5	-7.5	2.9	-1.6
155	4.5	-7.5	3.7	-1.6
156	4.5	-7.5	4.5	-1.6
157	4.5	-7.5	4.5	-1.6
158	4.5	-7.5	3.7	-1.6
159	4.5	-7.5	2.9	-1.6
160	4.5	-7.5	2.1	-1.6
161	4.5	-7.5	1.3	-1.6
162	4.5	-7.5	0.5	-1.6
163	4.5	-6.7	-0.3	-1.6
164	4.5	-5.9	-1.1	-1.6
165	4.5	-5.1	-1.9	-1.6
166	4.5	-4.3	-2.7	-1.6
167	4.5	-3.5	-3.5	-1.6
168	4.5	-2.7	-4.3	-1.6
169	4.5	-1.9	-5.1	-1.6
170	4.5	-1.1	-5.9	-1.6
171	4.5	-0.3	-6.7	-1.6
172	4.5	0.5	-7.5	-1.6
173	3.7	1.3	-7.5	-1.6
174	2.9	2.1	-6.7	-1.6
175	2.1	2.9	-5.9	-1.6
176	1.3	3.7	-5.1	-1.6
177	0.5	4.5	-4.3	-1.6
178	-0.3	4.5	-3.5	-1.6
179	-1.1	4.5	-2.7	-1.6
180	-1.9	4.5	-1.9	-1.6
181	-2.7	4.5	-1.1	-1.6
182	-3.5	4.5	-0.3	-1.6
183	-4.3	4.5	0.5	-1.6
184	-5.1	4.5	1.3	-1.6
185	-5.9	4.5	2.1	-1.6

186	-6.7	3.7	2.9	-1.6
187	-7.5	2.9	3.7	-1.6
188	-7.5	2.1	4.5	-1.6
189	-7.5	1.3	4.5	-1.6
190	-7.5	0.5	3.7	-1.6
191	-7.5	-0.3	2.9	-1.6
192	-7.5	-1.1	2.1	-1.6
193	-7.5	-1.9	1.3	-1.6
194	-7.5	-2.7	0.5	-1.6
195	-7.5	-3.5	-0.3	-1.6
196	-7.5	-4.3	-1.1	-1.6
197	-7.5	-5.1	-1.9	-1.6
198	-7.5	-5.9	-2.7	-1.6
199	-7.5	-6.7	-3.5	-1.6
200	-7.5	-7.5	-4.3	-1.6
201	-7.5	-7.5	-5.1	-1.6
202	-7.5	-7.5	-5.9	-1.6
203	-7.5	-7.5	-6.7	-1.6
204	-7.5	-7.5	-7.5	-1.6
205	-7.5	-7.5	-7.5	-1.6
206	-7.5	-7.5	-7.5	-1.6
207	-7.5	-7.5	-6.7	-1.6
208	-7.5	-7.5	-5.9	-1.6
209	-7.5	-7.5	-5.1	-1.6
210	-7.5	-6.7	-4.3	-1.6
211	-7.5	-5.9	-3.5	-1.6
212	-7.5	-5.1	-2.7	-1.6
213	-7.5	-4.3	-1.9	-1.6
214	-7.5	-3.5	-1.1	-1.6
215	-7.5	-2.7	-0.3	-1.6
216	-7.5	-1.9	0.5	-1.6
217	-7.5	-1.1	1.3	-1.6
218	-7.5	-0.3	2.1	-1.6
219	-6.7	0.5	2.9	-1.6
220	-5.9	1.3	3.7	-1.6
221	-5.1	2.1	4.5	-1.6
222	-4.3	2.9	4.5	-1.6
223	-3.5	3.7	3.7	-1.6
224	-2.7	4.5	2.9	-1.6
225	-1.9	4.5	2.1	-1.6

226	-1.1	4.5	1.3	-1.6
227	-0.3	4.5	0.5	-1.6
228	0.5	4.5	-0.3	-1.6
229	1.3	4.5	-1.1	-1.6
230	2.1	4.5	-1.9	-1.6
231	2.9	4.5	-2.7	-1.6
232	3.7	4.5	-3.5	-1.6
233	4.5	4.5	-4.3	-1.6
234	4.5	3.7	-5.1	-1.6
235	4.5	2.9	-5.9	-1.6
236	4.5	2.1	-6.7	-1.6
237	4.5	1.3	-7.5	-1.6
238	4.5	0.5	-7.5	-1.6
239	4.5	-0.3	-6.7	-1.6
240	4.5	-1.1	-6.7	-1.6
241	4.5	-1.9	-5.9	-1.6
242	4.5	-2.7	-5.1	-1.6
243	4.5	-3.5	-4.3	-1.6
244	4.5	-4.3	-3.5	-1.6
245	4.5	-5.1	-2.7	-1.6
246	4.5	-5.9	-1.9	-1.6
247	4.5	-6.7	-1.1	-1.6
248	4.5	-7.5	-0.3	-1.6
249	4.5	-7.5	0.5	-1.6
250	4.5	-7.5	1.3	-1.6
251	4.5	-7.5	2.1	-1.6
252	4.5	-7.5	2.9	-1.6
253	4.5	-7.5	3.7	-1.6
254	4.5	-7.5	4.5	-1.6
255	4.5	-7.5	4.5	-1.6
256	4.5	-7.5	3.7	-1.6
257	4.5	-7.5	2.9	-1.6
258	4.5	-7.5	2.1	-1.6
259	4.5	-6.7	1.3	-1.6
260	4.5	-5.9	0.5	-1.6
261	4.5	-5.1	-0.3	-1.6
262	4.5	-4.3	-1.1	-1.6
263	3.7	-3.5	-1.9	-1.6
264	2.9	-2.7	-2.7	-1.6
265	2.1	-1.9	-3.5	-1.6

266	1.3	-1.1	-4.3	-1.6
267	0.5	-0.3	-5.1	-1.6
268	-0.3	0.5	-5.9	-1.6
269	-1.1	1.3	-6.7	-1.6
270	-1.9	2.1	-7.5	-1.6
271	-2.7	2.9	-7.5	-1.6
272	-3.5	3.7	-6.7	-1.6
273	-4.3	4.5	-5.9	-1.6
274	-5.1	4.5	-5.1	-1.6
275	-5.9	4.5	-4.3	-1.6
276	-6.7	4.5	-3.5	-1.6
277	-7.5	4.5	-2.7	-1.6
278	-7.5	4.5	-1.9	-1.6
279	-7.5	4.5	-1.1	-1.6
280	-7.5	4.5	-0.3	-1.6
281	-7.5	4.5	0.5	-1.6
282	-7.5	3.7	1.3	-1.6
283	-7.5	2.9	2.1	-1.6
284	-7.5	2.1	2.9	-1.6
285	-7.5	1.3	3.7	-1.6
286	-7.5	0.5	4.5	-1.6
287	-7.5	-0.3	4.5	-1.6
288	-7.5	-1.1	3.7	-1.6
289	-7.5	-1.9	2.9	-1.6
290	-7.5	-2.7	2.1	-1.6
291	-7.5	-3.5	1.3	-1.6
292	-7.5	-4.3	0.5	-1.6
293	-7.5	-5.1	-0.3	-1.6
294	-7.5	-5.9	-1.1	-1.6
295	-7.5	-6.7	-1.9	-1.6
296	-7.5	-7.5	-2.7	-1.6
297	-7.5	-7.5	-3.5	-1.6
298	-7.5	-7.5	-4.3	-1.6
299	-7.5	-7.5	-5.1	-1.6
300	-7.5	-7.5	-5.9	-1.6
301	-7.5	-7.5	-6.7	-1.6
302	-7.5	-7.5	-7.5	-1.6
303	-7.5	-7.5	-7.5	-1.6
304	-7.5	-7.5	-7.5	-1.6
305	-7.5	-7.5	-6.7	-1.6

306	-7.5	-6.7	-5.9	-1.6
307	-7.5	-5.9	-5.1	-1.6
308	-7.5	-5.1	-4.3	-1.6
309	-6.7	-4.3	-3.5	-1.6
310	-5.9	-3.5	-2.7	-1.6
311	-5.1	-2.7	-1.9	-1.6
312	-4.3	-1.9	-1.1	-1.6
313	-3.5	-1.1	-0.3	-1.6
314	-2.7	-0.3	0.5	-1.6
315	-1.9	0.5	1.3	-1.6
316	-1.1	1.3	2.1	-1.6
317	-0.3	2.1	2.9	-1.6
318	0.5	2.9	3.7	-1.6
319	1.3	3.7	4.5	-1.6
320	2.1	4.5	4.5	-1.6
321	2.9	4.5	3.7	-1.6
322	3.7	4.5	2.9	-1.6
323	4.5	4.5	2.1	-1.6
324	4.5	4.5	1.3	-1.6
325	4.5	4.5	0.5	-1.6
326	4.5	4.5	-0.3	-1.6
327	4.5	4.5	-1.1	-1.6
328	4.5	4.5	-1.9	-1.6
329	4.5	4.5	-2.7	-1.6
330	4.5	3.7	-3.5	-1.6
331	4.5	2.9	-4.3	-1.6
332	4.5	2.1	-5.1	-1.6
333	4.5	1.3	-5.9	-1.6
334	4.5	0.5	-6.7	-1.6
335	4.5	-0.3	-7.5	-1.6
336	4.5	-1.1	-7.5	-1.6
337	4.5	-1.9	-7.5	-1.6
338	4.5	-2.7	-6.7	-1.6
339	4.5	-3.5	-5.9	-1.6
340	4.5	-4.3	-5.1	-1.6
341	4.5	-5.1	-4.3	-1.6
342	4.5	-5.9	-3.5	-1.6
343	4.5	-6.7	-2.7	-1.6
344	4.5	-7.5	-1.9	-1.6
345	4.5	-7.5	-1.1	-1.6

346	4.5	-7.5	-0.3	-1.6
347	4.5	-7.5	0.5	-1.6
348	4.5	-7.5	1.3	-1.6
349	4.5	-7.5	2.1	-1.6
350	4.5	-7.5	2.9	-1.6
351	4.5	-7.5	3.7	-1.6
352	4.5	-7.5	4.5	-1.6
353	3.7	-7.5	4.5	-1.6
354	2.9	-7.5	3.7	-1.6
355	2.1	-6.7	2.9	-1.6
356	1.3	-5.9	2.1	-1.6
357	0.5	-5.1	1.3	-1.6
358	-0.3	-4.3	0.5	-1.6
359	-1.1	-3.5	-0.3	-1.6
360	-1.9	-2.7	-1.1	-1.6
361	-2.7	-1.9	-1.9	-1.6
362	-3.5	-1.1	-2.7	-1.6
363	-4.3	-0.3	-3.5	-1.6
364	-5.1	0.5	-4.3	-1.6
365	-5.9	1.3	-5.1	-1.6
366	-6.7	2.1	-5.9	-1.6
367	-7.5	2.9	-6.7	-1.6
368	-7.5	3.7	-7.5	-1.6
369	-7.5	4.5	-7.5	-1.6
370	-7.5	4.5	-6.7	-1.6
371	-7.5	4.5	-5.9	-1.6
372	-7.5	4.5	-5.1	-1.6
373	-7.5	4.5	-4.3	-1.6
374	-7.5	4.5	-3.5	-1.6
375	-7.5	4.5	-2.7	-1.6
376	-7.5	4.5	-1.9	-1.6
377	-7.5	4.5	-1.1	-1.6
378	-7.5	3.7	-0.3	-1.6
379	-7.5	2.9	0.5	-1.6
380	-7.5	2.1	1.3	-1.6
381	-7.5	1.3	2.1	-1.6
382	-7.5	0.5	2.9	-1.6
383	-7.5	-0.3	3.7	-1.6
384	-7.5	-1.1	4.5	-1.6
385	-7.5	-1.9	4.5	-1.6

386	-7.5	-2.7	3.7	-1.6
387	-7.5	-3.5	2.9	-1.6
388	-7.5	-4.3	2.1	-1.6
389	-7.5	-5.1	1.3	-1.6
390	-7.5	-5.9	0.5	-1.6
391	-7.5	-6.7	-0.3	-1.6
392	-7.5	-7.5	-1.1	-1.6
393	-7.5	-7.5	-1.9	-1.6
394	-7.5	-7.5	-2.7	-1.6
395	-7.5	-7.5	-3.5	-1.6
396	-7.5	-7.5	-4.3	-1.6
397	-7.5	-7.5	-5.1	-1.6
398	-7.5	-7.5	-5.9	-1.6
399	-6.7	-7.5	-6.7	-1.6
400	-5.9	-7.5	-7.5	-1.6
401	-5.1	-7.5	-7.5	-1.6
402	-4.3	-6.7	-6.7	-1.6
403	-3.5	-5.9	-6.7	-1.6
404	-2.7	-5.1	-5.9	-1.6
405	-1.9	-4.3	-5.1	-1.6
406	-1.1	-3.5	-4.3	-1.6
407	-0.3	-2.7	-3.5	-1.6
408	0.5	-1.9	-2.7	-1.6
409	1.3	-1.1	-1.9	-1.6
410	2.1	-0.3	-1.1	-1.6
411	2.9	0.5	-0.3	-1.6
412	3.7	1.3	0.5	-1.6
413	4.5	2.1	1.3	-1.6
414	4.5	2.9	2.1	-1.6
415	4.5	3.7	2.9	-1.6
416	4.5	4.5	3.7	-1.6
417	4.5	4.5	4.5	-1.6
418	4.5	4.5	4.5	-1.6
419	4.5	4.5	3.7	-1.6
420	4.5	4.5	2.9	-1.6
421	4.5	4.5	2.1	-1.6
422	4.5	4.5	1.3	-1.6
423	4.5	4.5	0.5	-1.6
424	4.5	4.5	-0.3	-1.6
425	4.5	4.5	-1.1	-1.6

426	4.5	3.7	-1.9	-1.6
427	4.5	2.9	-2.7	-1.6
428	4.5	2.1	-3.5	-1.6
429	4.5	1.3	-4.3	-1.6
430	4.5	0.5	-5.1	-1.6
431	4.5	-0.3	-5.9	-1.6
432	4.5	-1.1	-6.7	-1.6
433	4.5	-1.9	-7.5	-1.6
434	4.5	-2.7	-7.5	-1.6
435	4.5	-3.5	-6.7	-1.6
436	4.5	-4.3	-5.9	-1.6
437	4.5	-5.1	-5.1	-1.6
438	4.5	-5.9	-4.3	-1.6
439	4.5	-6.7	-3.5	-1.6
440	4.5	-7.5	-2.7	-1.6
441	4.5	-7.5	-1.9	-1.6
442	4.5	-7.5	-1.1	-1.6
443	3.7	-7.5	-0.3	-1.6
444	2.9	-7.5	0.5	-1.6
445	2.1	-7.5	1.3	-1.6
446	1.3	-7.5	2.1	-1.6
447	0.5	-7.5	2.9	-1.6
448	-0.3	-7.5	3.7	-1.6
449	-1.1	-7.5	4.5	-1.6
450	-1.9	-7.5	4.5	-1.6
451	-2.7	-6.7	3.7	-1.6
452	-3.5	-5.9	2.9	-1.6
453	-4.3	-5.1	2.1	-1.6
454	-5.1	-4.3	1.3	-1.6
455	-5.9	-3.5	0.5	-1.6
456	-6.7	-2.7	-0.3	-1.6
457	-7.5	-1.9	-1.1	-1.6
458	-7.5	-1.1	-1.9	-1.6
459	-7.5	-0.3	-2.7	-1.6
460	-7.5	0.5	-3.5	-1.6
461	-7.5	1.3	-4.3	-1.6
462	-7.5	2.1	-5.1	-1.6
463	-7.5	2.9	-5.9	-1.6
464	-7.5	3.7	-6.7	-1.6
465	-7.5	4.5	-7.5	-1.6

466	-7.5	4.5	-7.5	-1.6
467	-7.5	4.5	-7.5	-1.6
468	-7.5	4.5	-6.7	-1.6
469	-7.5	4.5	-5.9	-1.6
470	-7.5	4.5	-5.1	-1.6
471	-7.5	4.5	-4.3	-1.6
472	-7.5	4.5	-3.5	-1.6
473	-7.5	4.5	-2.7	-1.6
474	-7.5	3.7	-1.9	-1.6
475	-7.5	2.9	-1.1	-1.6
476	-7.5	2.1	-0.3	-1.6
477	-7.5	1.3	0.5	-1.6
478	-7.5	0.5	1.3	-1.6
479	-7.5	-0.3	2.1	-1.6
480	-7.5	-1.1	2.9	-1.6
481	-7.5	-1.9	3.7	-1.6
482	-7.5	-2.7	4.5	-1.6
483	-7.5	-3.5	4.5	-1.6
484	-7.5	-4.3	3.7	-1.6
485	-7.5	-5.1	2.9	-1.6
486	-7.5	-5.9	2.1	-1.6
487	-7.5	-6.7	1.3	-1.6
488	-7.5	-7.5	0.5	-1.6
489	-6.7	-7.5	-0.3	-1.6
490	-5.9	-7.5	-1.1	-1.6
491	-5.1	-7.5	-1.9	-1.6
492	-4.3	-7.5	-2.7	-1.6
493	-3.5	-7.5	-3.5	-1.6
494	-2.7	-7.5	-4.3	-1.6
495	-1.9	-7.5	-5.1	-1.6
496	-1.1	-7.5	-5.9	-1.6
497	-0.3	-7.5	-6.7	-1.6
498	0.5	-6.7	-7.5	-1.6
499	1.3	-5.9	-7.5	-1.6
500	2.1	-5.1	-6.7	-1.6
501	2.9	-4.3	-5.9	-1.6
502	3.7	-3.5	-5.1	-1.6
503	4.5	-2.7	-4.3	-1.6
504	4.5	-1.9	-3.5	-1.6
505	4.5	-1.1	-2.7	-1.6

506	4.5	-0.3	-1.9	-1.6
507	4.5	0.5	-1.1	-1.6
508	4.5	1.3	-0.3	-1.6
509	4.5	2.1	0.5	-1.6
510	4.5	2.9	1.3	-1.6
511	4.5	3.7	2.1	-1.6
512	4.5	4.5	2.9	-1.6
513	4.5	4.5	3.7	-1.6
514	4.5	4.5	4.5	-1.6
515	4.5	4.5	4.5	-1.6
516	4.5	4.5	3.7	-1.6
517	4.5	4.5	2.9	-1.6
518	4.5	4.5	2.1	-1.6
519	4.5	4.5	1.3	-1.6
520	4.5	4.5	0.5	-1.6
521	4.5	4.5	-0.3	-1.6
522	4.5	3.7	-1.1	-1.6
523	4.5	2.9	-1.9	-1.6
524	4.5	2.1	-2.7	-1.6
525	4.5	1.3	-3.5	-1.6
526	4.5	0.5	-4.3	-1.6
527	4.5	-0.3	-5.1	-1.6
528	4.5	-1.1	-5.9	-1.6
529	4.5	-1.9	-6.7	-1.6
530	4.5	-2.7	-7.5	-1.6
531	4.5	-3.5	-7.5	-1.6
532	4.5	-4.3	-7.5	-1.6
533	3.7	-5.1	-6.7	-1.6
534	2.9	-5.9	-5.9	-1.6
535	2.1	-6.7	-5.1	-1.6
536	1.3	-7.5	-4.3	-1.6
537	0.5	-7.5	-3.5	-1.6
538	-0.3	-7.5	-2.7	-1.6
539	-1.1	-7.5	-1.9	-1.6
540	-1.9	-7.5	-1.1	-1.6
541	-2.7	-7.5	-0.3	-1.6
542	-3.5	-7.5	0.5	-1.6
543	-4.3	-7.5	1.3	-1.6
544	-5.1	-7.5	2.1	-1.6
545	-5.9	-7.5	2.9	-1.6

546	-6.7	-7.5	3.7	-1.6
547	-7.5	-6.7	4.5	-1.6
548	-7.5	-5.9	4.5	-1.6
549	-7.5	-5.1	3.7	-1.6
550	-7.5	-4.3	2.9	-1.6
551	-7.5	-3.5	2.1	-1.6
552	-7.5	-2.7	1.3	-1.6
553	-7.5	-1.9	0.5	-1.6
554	-7.5	-1.1	-0.3	-1.6
555	-7.5	-0.3	-1.1	-1.6
556	-7.5	0.5	-1.9	-1.6
557	-7.5	1.3	-2.7	-1.6
558	-7.5	2.1	-3.5	-1.6
559	-7.5	2.9	-4.3	-1.6
560	-7.5	3.7	-5.1	-1.6
561	-7.5	4.5	-5.9	-1.6
562	-7.5	4.5	-6.7	-1.6
563	-7.5	4.5	-7.5	-1.6
564	-7.5	4.5	-7.5	-1.6
565	-7.5	4.5	-6.7	-1.6
566	-7.5	4.5	-5.9	-1.6
567	-7.5	4.5	-5.1	-1.6
568	-7.5	4.5	-4.3	-1.6
569	-7.5	4.5	-3.5	-1.6
570	-7.5	3.7	-2.7	-1.6
571	-7.5	2.9	-1.9	-1.6
572	-7.5	2.1	-1.1	-1.6
573	-7.5	1.3	-0.3	-1.6
574	-7.5	0.5	0.5	-1.6
575	-7.5	-0.3	1.3	-1.6
576	-7.5	-1.1	2.1	-1.6
577	-7.5	-1.9	2.9	-1.6
578	-7.5	-2.7	3.7	-1.6
579	-6.7	-3.5	4.5	-1.6
580	-5.9	-4.3	4.5	-1.6
581	-5.1	-5.1	3.7	-1.6
582	-4.3	-5.9	2.9	-1.6
583	-3.5	-6.7	2.1	-1.6
584	-2.7	-7.5	1.3	-1.6
585	-1.9	-7.5	0.5	-1.6

586	-1.1	-7.5	-0.3	-1.6
587	-0.3	-7.5	-1.1	-1.6
588	0.5	-7.5	-1.9	-1.6
589	1.3	-7.5	-2.7	-1.6
590	2.1	-7.5	-3.5	-1.6
591	2.9	-7.5	-4.3	-1.6
592	3.7	-7.5	-5.1	-1.6
593	4.5	-7.5	-5.9	-1.6
594	4.5	-6.7	-6.7	-1.6
595	4.5	-5.9	-7.5	-1.6
596	4.5	-5.1	-7.5	-1.6
597	4.5	-4.3	-7.5	-1.6
598	4.5	-3.5	-6.7	-1.6
599	4.5	-2.7	-5.9	-1.6
600	4.5	-1.9	-5.1	-1.6
601	4.5	-1.1	-4.3	-1.6
602	4.5	-0.3	-3.5	-1.6
603	4.5	0.5	-2.7	-1.6
604	4.5	1.3	-1.9	-1.6
605	4.5	2.1	-1.1	-1.6
606	4.5	2.9	-0.3	-1.6
607	4.5	3.7	0.5	-1.6
608	4.5	4.5	1.3	-1.6
609	4.5	4.5	2.1	-1.6
610	4.5	4.5	2.9	-1.6
611	4.5	4.5	3.7	-1.6
612	4.5	4.5	4.5	-1.6
613	4.5	4.5	4.5	-1.6
614	4.5	4.5	3.7	-1.6
615	4.5	4.5	2.9	-1.6
616	4.5	4.5	2.1	-1.6
617	4.5	4.5	1.3	-1.6
618	4.5	3.7	0.5	-1.6
619	4.5	2.9	-0.3	-1.6
620	4.5	2.1	-1.1	-1.6
621	4.5	1.3	-1.9	-1.6
622	4.5	0.5	-2.7	-1.6
623	3.7	-0.3	-3.5	-1.6
624	2.9	-1.1	-4.3	-1.6
625	2.1	-1.9	-5.1	-1.6

626	1.3	-2.7	-5.9	-1.6
627	0.5	-3.5	-6.7	-1.6
628	-0.3	-4.3	-7.5	-1.6
629	-1.1	-5.1	-7.5	-1.6
630	-1.9	-5.9	-7.5	-1.6
631	-2.7	-6.7	-6.7	-1.6
632	-3.5	-7.5	-5.9	-1.6
633	-4.3	-7.5	-5.1	-1.6
634	-5.1	-7.5	-4.3	-1.6
635	-5.9	-7.5	-3.5	-1.6
636	-6.7	-7.5	-2.7	-1.6
637	-7.5	-7.5	-1.9	-1.6
638	-7.5	-7.5	-1.1	-1.6
639	-7.5	-7.5	-0.3	-1.6
640	-7.5	-7.5	0.5	-1.6
641	-7.5	-7.5	1.3	-1.6
642	-7.5	-7.5	2.1	-1.6
643	-7.5	-6.7	2.9	-1.6
644	-7.5	-5.9	3.7	-1.6
645	-7.5	-5.1	4.5	-1.6
646	-7.5	-4.3	4.5	-1.6
647	-7.5	-3.5	3.7	-1.6
648	-7.5	-2.7	2.9	-1.6
649	-7.5	-1.9	2.1	-1.6
650	-7.5	-1.1	1.3	-1.6
651	-7.5	-0.3	0.5	-1.6
652	-7.5	0.5	-0.3	-1.6
653	-7.5	1.3	-1.1	-1.6
654	-7.5	2.1	-1.9	-1.6
655	-7.5	2.9	-2.7	-1.6
656	-7.5	3.7	-3.5	-1.6
657	-7.5	4.5	-4.3	-1.6
658	-7.5	4.5	-5.1	-1.6
659	-7.5	4.5	-5.9	-1.6
660	-7.5	4.5	-6.7	-1.6
661	-7.5	4.5	-7.5	-1.6
662	-7.5	4.5	-7.5	-1.6
663	-7.5	4.5	-7.5	-1.6
664	-7.5	4.5	-6.7	-1.6
665	-7.5	4.5	-5.9	-1.6

666	-7.5	3.7	-5.1	-1.6
667	-7.5	2.9	-4.3	-1.6
668	-7.5	2.1	-3.5	-1.6
669	-6.7	1.3	-2.7	-1.6
670	-5.9	0.5	-1.9	-1.6
671	-5.1	-0.3	-1.1	-1.6
672	-4.3	-1.1	-0.3	-1.6
673	-3.5	-1.9	0.5	-1.6
674	-2.7	-2.7	1.3	-1.6
675	-1.9	-3.5	2.1	-1.6
676	-1.1	-4.3	2.9	-1.6
677	-0.3	-5.1	3.7	-1.6
678	0.5	-5.9	4.5	-1.6
679	1.3	-6.7	4.5	-1.6
680	2.1	-7.5	3.7	-1.6
681	2.9	-7.5	2.9	-1.6
682	3.7	-7.5	2.1	-1.6
683	4.5	-7.5	1.3	-1.6
684	4.5	-7.5	0.5	-1.6
685	4.5	-7.5	-0.3	-1.6
686	4.5	-7.5	-1.1	-1.6
687	4.5	-7.5	-1.9	-1.6
688	4.5	-7.5	-2.7	-1.6
689	4.5	-7.5	-3.5	-1.6
690	4.5	-6.7	-4.3	-1.6
691	4.5	-5.9	-5.1	-1.6
692	4.5	-5.1	-5.9	-1.6
693	4.5	-4.3	-6.7	-1.6
694	4.5	-3.5	-7.5	-1.6
695	4.5	-2.7	-7.5	-1.6
696	4.5	-1.9	-7.5	-1.6
697	4.5	-1.1	-6.7	-1.6
698	4.5	-0.3	-5.9	-1.6
699	4.5	0.5	-5.1	-1.6
700	4.5	1.3	-4.3	-1.6
701	4.5	2.1	-3.5	-1.6
702	4.5	2.9	-2.7	-1.6
703	4.5	3.7	-1.9	-1.6
704	4.5	4.5	-1.1	-1.6
705	4.5	4.5	-0.3	-1.6

706	4.5	4.5	0.5	-1.6
707	4.5	4.5	1.3	-1.6
708	4.5	4.5	2.1	-1.6
709	4.5	4.5	2.9	-1.6
710	4.5	4.5	3.7	-1.6
711	4.5	4.5	4.5	-1.6
712	4.5	4.5	4.5	-1.6
713	3.7	3.7	3.7	-1.6
714	2.9	2.9	2.9	-1.6
715	2.1	2.1	2.1	-1.6
716	1.3	1.3	1.3	-1.6
717	0.5	0.5	0.5	-1.6
718	-0.3	-0.3	-0.3	-1.6
719	-1.1	-1.1	-1.1	-1.6
720	-1.9	-1.9	-1.9	-1.6

8
