# Supplementary material for: Experimental evidence demonstrating how freeze-thaw patterns affect spoilage of perishable cached food
Source: PLoS One. 2025 Apr 4;20(4):e0319043. doi: 10.1371/journal.pone.0319043 (PMC11970643; doi:10.1371/journal.pone.0319043)
Supplement: S5 Table — Each freezer was dedicated to simulating either the average freeze-thaw treatment (freezing and thawing rate of 0.8 °C/hr), mild freeze treatment (freezing rate of 0.4 °C/hr; thawing rate of 0.8 °C/hr), mild thaw treatment (freezing rate of 0.8 °C/hr; thawing rate of 0.4 °C/hr), low temperature control (held at −4.9 °C, no freeze-thaw events), or high temperature control (held at 1.1 °C, no freeze-thaw events). All simulations were 720 hours long and each treatment group had eight freeze-thaw events. (PDF) [file pone.0319043.s005.pdf]

S5 Table. Hourly programming schedule for all freezers used in experiment 3 to test the predictions of the '*thaw intensity hypothesis*' and '*freeze intensity hypothesis*'. Each freezer was dedicated to simulating either the average freeze-thaw treatment (freezing and thawing rate of 0.8°C/hr), mild freeze treatment (freezing rate of 0.4°C/hr; thawing rate of 0.8°C/hr), mild thaw treatment (freezing rate of 0.8°C/hr; thawing rate of 0.4°C/hr), low temperature control (held at -4.9°C, no freeze-thaw events), or high temperature control (held at 1.1°C, no freeze-thaw events). All simulations were 720 hours long and each treatment group had eight freeze-thaw events.

Hour	Average freeze-thaw (°C)	Mild freeze (°C)	Mild thaw (°C)	Low temperature control (°C)	High temperature control (°C)
1	1.3	1.3	-0.3	-4.9	1.1
2	0.5	0.5	-0.7	-4.9	1.1
3	-0.3	-0.3	-1.1	-4.9	1.1
4	-1.1	-1.1	-1.5	-4.9	1.1
5	-1.9	-1.9	-1.9	-4.9	1.1
6	-2.7	-2.3	-2.7	-4.9	1.1
7	-3.5	-2.7	-3.5	-4.9	1.1
8	-4.3	-3.1	-4.3	-4.9	1.1
9	-5.1	-3.5	-5.1	-4.9	1.1
10	-5.9	-3.9	-5.9	-4.9	1.1
11	-6.7	-4.3	-6.7	-4.9	1.1
12	-6.7	-4.3	-6.7	-4.9	1.1
13	-6.7	-4.3	-6.7	-4.9	1.1
14	-6.7	-4.3	-6.7	-4.9	1.1
15	-6.7	-4.3	-6.7	-4.9	1.1
16	-6.7	-4.3	-6.7	-4.9	1.1
17	-6.7	-4.3	-6.7	-4.9	1.1
18	-6.7	-4.3	-6.7	-4.9	1.1
19	-6.7	-4.3	-6.7	-4.9	1.1
20	-6.7	-4.3	-6.7	-4.9	1.1
21	-6.7	-4.3	-6.7	-4.9	1.1
22	-6.7	-4.3	-6.7	-4.9	1.1
23	-6.7	-4.3	-6.7	-4.9	1.1

24	-6.7	-4.3	-6.7	-4.9	1.1
25	-6.7	-4.3	-6.7	-4.9	1.1
26	-6.7	-4.3	-6.7	-4.9	1.1
27	-6.7	-4.3	-6.7	-4.9	1.1
28	-6.7	-4.3	-6.7	-4.9	1.1
29	-6.7	-4.3	-6.7	-4.9	1.1
30	-6.7	-4.3	-6.7	-4.9	1.1
31	-6.7	-4.3	-6.7	-4.9	1.1
32	-6.7	-4.3	-6.7	-4.9	1.1
33	-6.7	-4.3	-6.7	-4.9	1.1
34	-6.7	-4.3	-6.7	-4.9	1.1
35	-6.7	-4.3	-6.7	-4.9	1.1
36	-6.7	-4.3	-6.7	-4.9	1.1
37	-6.7	-4.3	-6.7	-4.9	1.1
38	-6.7	-4.3	-6.7	-4.9	1.1
39	-6.7	-4.3	-6.7	-4.9	1.1
40	-6.7	-4.3	-6.7	-4.9	1.1
41	-6.7	-4.3	-6.7	-4.9	1.1
42	-6.7	-4.3	-6.7	-4.9	1.1
43	-6.7	-4.3	-6.7	-4.9	1.1
44	-6.7	-4.3	-6.7	-4.9	1.1
45	-6.7	-4.3	-6.7	-4.9	1.1
46	-6.7	-4.3	-6.7	-4.9	1.1
47	-6.7	-4.3	-6.7	-4.9	1.1
48	-6.7	-4.3	-6.7	-4.9	1.1
49	-6.7	-4.3	-6.7	-4.9	1.1
50	-6.7	-4.3	-6.7	-4.9	1.1
51	-6.7	-4.3	-6.7	-4.9	1.1
52	-6.7	-4.3	-6.7	-4.9	1.1
53	-6.7	-4.3	-6.7	-4.9	1.1
54	-6.7	-4.3	-6.7	-4.9	1.1
55	-6.7	-4.3	-6.7	-4.9	1.1
56	-6.7	-4.3	-6.7	-4.9	1.1
57	-6.7	-4.3	-6.7	-4.9	1.1
58	-6.7	-4.3	-6.7	-4.9	1.1
59	-6.7	-4.3	-6.7	-4.9	1.1
60	-6.7	-4.3	-6.7	-4.9	1.1
61	-6.7	-4.3	-6.7	-4.9	1.1
62	-6.7	-4.3	-6.7	-4.9	1.1
63	-6.7	-4.3	-6.7	-4.9	1.1

64	-6.7	-4.3	-6.7	-4.9	1.1
65	-6.7	-4.3	-6.7	-4.9	1.1
66	-6.7	-4.3	-6.7	-4.9	1.1
67	-6.7	-4.3	-6.7	-4.9	1.1
68	-6.7	-4.3	-6.7	-4.9	1.1
69	-6.7	-4.3	-6.7	-4.9	1.1
70	-6.7	-4.3	-6.7	-4.9	1.1
71	-6.7	-4.3	-6.7	-4.9	1.1
72	-5.9	-3.9	-5.9	-4.9	1.1
73	-5.1	-3.5	-5.1	-4.9	1.1
74	-4.3	-3.1	-4.3	-4.9	1.1
75	-3.5	-2.7	-3.5	-4.9	1.1
76	-2.7	-2.3	-2.7	-4.9	1.1
77	-1.9	-1.9	-1.9	-4.9	1.1
78	-1.1	-1.1	-1.5	-4.9	1.1
79	-0.3	-0.3	-1.1	-4.9	1.1
80	0.5	0.5	-0.7	-4.9	1.1
81	1.3	1.3	-0.3	-4.9	1.1
82	2.1	2.1	0.1	-4.9	1.1
83	2.9	2.9	0.5	-4.9	1.1
84	3.7	3.7	0.9	-4.9	1.1
85	4.5	4.5	1.3	-4.9	1.1
86	4.5	4.5	1.3	-4.9	1.1
87	3.7	3.7	0.9	-4.9	1.1
88	2.9	2.9	0.5	-4.9	1.1
89	2.1	2.1	0.1	-4.9	1.1
90	1.3	1.3	-0.3	-4.9	1.1
91	0.5	0.5	-0.7	-4.9	1.1
92	-0.3	-0.3	-1.1	-4.9	1.1
93	-1.1	-1.1	-1.5	-4.9	1.1
94	-1.9	-1.9	-1.9	-4.9	1.1
95	-2.7	-2.3	-2.7	-4.9	1.1
96	-3.5	-2.7	-3.5	-4.9	1.1
97	-4.3	-3.1	-4.3	-4.9	1.1
98	-5.1	-3.5	-5.1	-4.9	1.1
99	-5.9	-3.9	-5.9	-4.9	1.1
100	-6.7	-4.3	-6.7	-4.9	1.1
101	-6.7	-4.3	-6.7	-4.9	1.1
102	-6.7	-4.3	-6.7	-4.9	1.1
103	-6.7	-4.3	-6.7	-4.9	1.1

104	-6.7	-4.3	-6.7	-4.9	1.1
105	-6.7	-4.3	-6.7	-4.9	1.1
106	-6.7	-4.3	-6.7	-4.9	1.1
107	-6.7	-4.3	-6.7	-4.9	1.1
108	-6.7	-4.3	-6.7	-4.9	1.1
109	-6.7	-4.3	-6.7	-4.9	1.1
110	-6.7	-4.3	-6.7	-4.9	1.1
111	-6.7	-4.3	-6.7	-4.9	1.1
112	-6.7	-4.3	-6.7	-4.9	1.1
113	-6.7	-4.3	-6.7	-4.9	1.1
114	-6.7	-4.3	-6.7	-4.9	1.1
115	-6.7	-4.3	-6.7	-4.9	1.1
116	-6.7	-4.3	-6.7	-4.9	1.1
117	-6.7	-4.3	-6.7	-4.9	1.1
118	-6.7	-4.3	-6.7	-4.9	1.1
119	-6.7	-4.3	-6.7	-4.9	1.1
120	-6.7	-4.3	-6.7	-4.9	1.1
121	-6.7	-4.3	-6.7	-4.9	1.1
122	-6.7	-4.3	-6.7	-4.9	1.1
123	-6.7	-4.3	-6.7	-4.9	1.1
124	-6.7	-4.3	-6.7	-4.9	1.1
125	-6.7	-4.3	-6.7	-4.9	1.1
126	-6.7	-4.3	-6.7	-4.9	1.1
127	-6.7	-4.3	-6.7	-4.9	1.1
128	-6.7	-4.3	-6.7	-4.9	1.1
129	-6.7	-4.3	-6.7	-4.9	1.1
130	-6.7	-4.3	-6.7	-4.9	1.1
131	-6.7	-4.3	-6.7	-4.9	1.1
132	-6.7	-4.3	-6.7	-4.9	1.1
133	-6.7	-4.3	-6.7	-4.9	1.1
134	-6.7	-4.3	-6.7	-4.9	1.1
135	-6.7	-4.3	-6.7	-4.9	1.1
136	-6.7	-4.3	-6.7	-4.9	1.1
137	-6.7	-4.3	-6.7	-4.9	1.1
138	-6.7	-4.3	-6.7	-4.9	1.1
139	-6.7	-4.3	-6.7	-4.9	1.1
140	-6.7	-4.3	-6.7	-4.9	1.1
141	-6.7	-4.3	-6.7	-4.9	1.1
142	-6.7	-4.3	-6.7	-4.9	1.1
143	-6.7	-4.3	-6.7	-4.9	1.1

144	-6.7	-4.3	-6.7	-4.9	1.1
145	-6.7	-4.3	-6.7	-4.9	1.1
146	-6.7	-4.3	-6.7	-4.9	1.1
147	-6.7	-4.3	-6.7	-4.9	1.1
148	-6.7	-4.3	-6.7	-4.9	1.1
149	-6.7	-4.3	-6.7	-4.9	1.1
150	-6.7	-4.3	-6.7	-4.9	1.1
151	-6.7	-4.3	-6.7	-4.9	1.1
152	-6.7	-4.3	-6.7	-4.9	1.1
153	-6.7	-4.3	-6.7	-4.9	1.1
154	-6.7	-4.3	-6.7	-4.9	1.1
155	-6.7	-4.3	-6.7	-4.9	1.1
156	-6.7	-4.3	-6.7	-4.9	1.1
157	-6.7	-4.3	-6.7	-4.9	1.1
158	-6.7	-4.3	-6.7	-4.9	1.1
159	-6.7	-4.3	-6.7	-4.9	1.1
160	-6.7	-4.3	-6.7	-4.9	1.1
161	-5.9	-3.9	-5.9	-4.9	1.1
162	-5.1	-3.5	-5.1	-4.9	1.1
163	-4.3	-3.1	-4.3	-4.9	1.1
164	-3.5	-2.7	-3.5	-4.9	1.1
165	-2.7	-2.3	-2.7	-4.9	1.1
166	-1.9	-1.9	-1.9	-4.9	1.1
167	-1.1	-1.1	-1.5	-4.9	1.1
168	-0.3	-0.3	-1.1	-4.9	1.1
169	0.5	0.5	-0.7	-4.9	1.1
170	1.3	1.3	-0.3	-4.9	1.1
171	2.1	2.1	0.1	-4.9	1.1
172	2.9	2.9	0.5	-4.9	1.1
173	3.7	3.7	0.9	-4.9	1.1
174	4.5	4.5	1.3	-4.9	1.1
175	4.5	4.5	1.3	-4.9	1.1
176	3.7	3.7	0.9	-4.9	1.1
177	2.9	2.9	0.5	-4.9	1.1
178	2.1	2.1	0.1	-4.9	1.1
179	1.3	1.3	-0.3	-4.9	1.1
180	0.5	0.5	-0.7	-4.9	1.1
181	-0.3	-0.3	-1.1	-4.9	1.1
182	-1.1	-1.1	-1.5	-4.9	1.1
183	-1.9	-1.9	-1.9	-4.9	1.1

184	-2.7	-2.3	-2.7	-4.9	1.1
185	-3.5	-2.7	-3.5	-4.9	1.1
186	-4.3	-3.1	-4.3	-4.9	1.1
187	-5.1	-3.5	-5.1	-4.9	1.1
188	-5.9	-3.9	-5.9	-4.9	1.1
189	-6.7	-4.3	-6.7	-4.9	1.1
190	-6.7	-4.3	-6.7	-4.9	1.1
191	-6.7	-4.3	-6.7	-4.9	1.1
192	-6.7	-4.3	-6.7	-4.9	1.1
193	-6.7	-4.3	-6.7	-4.9	1.1
194	-6.7	-4.3	-6.7	-4.9	1.1
195	-6.7	-4.3	-6.7	-4.9	1.1
196	-6.7	-4.3	-6.7	-4.9	1.1
197	-6.7	-4.3	-6.7	-4.9	1.1
198	-6.7	-4.3	-6.7	-4.9	1.1
199	-6.7	-4.3	-6.7	-4.9	1.1
200	-6.7	-4.3	-6.7	-4.9	1.1
201	-6.7	-4.3	-6.7	-4.9	1.1
202	-6.7	-4.3	-6.7	-4.9	1.1
203	-6.7	-4.3	-6.7	-4.9	1.1
204	-6.7	-4.3	-6.7	-4.9	1.1
205	-6.7	-4.3	-6.7	-4.9	1.1
206	-6.7	-4.3	-6.7	-4.9	1.1
207	-6.7	-4.3	-6.7	-4.9	1.1
208	-6.7	-4.3	-6.7	-4.9	1.1
209	-6.7	-4.3	-6.7	-4.9	1.1
210	-6.7	-4.3	-6.7	-4.9	1.1
211	-6.7	-4.3	-6.7	-4.9	1.1
212	-6.7	-4.3	-6.7	-4.9	1.1
213	-6.7	-4.3	-6.7	-4.9	1.1
214	-6.7	-4.3	-6.7	-4.9	1.1
215	-6.7	-4.3	-6.7	-4.9	1.1
216	-6.7	-4.3	-6.7	-4.9	1.1
217	-6.7	-4.3	-6.7	-4.9	1.1
218	-6.7	-4.3	-6.7	-4.9	1.1
219	-6.7	-4.3	-6.7	-4.9	1.1
220	-6.7	-4.3	-6.7	-4.9	1.1
221	-6.7	-4.3	-6.7	-4.9	1.1
222	-6.7	-4.3	-6.7	-4.9	1.1
223	-6.7	-4.3	-6.7	-4.9	1.1

224	-6.7	-4.3	-6.7	-4.9	1.1
225	-6.7	-4.3	-6.7	-4.9	1.1
226	-6.7	-4.3	-6.7	-4.9	1.1
227	-6.7	-4.3	-6.7	-4.9	1.1
228	-6.7	-4.3	-6.7	-4.9	1.1
229	-6.7	-4.3	-6.7	-4.9	1.1
230	-6.7	-4.3	-6.7	-4.9	1.1
231	-6.7	-4.3	-6.7	-4.9	1.1
232	-6.7	-4.3	-6.7	-4.9	1.1
233	-6.7	-4.3	-6.7	-4.9	1.1
234	-6.7	-4.3	-6.7	-4.9	1.1
235	-6.7	-4.3	-6.7	-4.9	1.1
236	-6.7	-4.3	-6.7	-4.9	1.1
237	-6.7	-4.3	-6.7	-4.9	1.1
238	-6.7	-4.3	-6.7	-4.9	1.1
239	-6.7	-4.3	-6.7	-4.9	1.1
240	-6.7	-4.3	-6.7	-4.9	1.1
241	-6.7	-4.3	-6.7	-4.9	1.1
242	-6.7	-4.3	-6.7	-4.9	1.1
243	-6.7	-4.3	-6.7	-4.9	1.1
244	-6.7	-4.3	-6.7	-4.9	1.1
245	-6.7	-4.3	-6.7	-4.9	1.1
246	-6.7	-4.3	-6.7	-4.9	1.1
247	-6.7	-4.3	-6.7	-4.9	1.1
248	-6.7	-4.3	-6.7	-4.9	1.1
249	-6.7	-4.3	-6.7	-4.9	1.1
250	-5.9	-3.9	-5.9	-4.9	1.1
251	-5.1	-3.5	-5.1	-4.9	1.1
252	-4.3	-3.1	-4.3	-4.9	1.1
253	-3.5	-2.7	-3.5	-4.9	1.1
254	-2.7	-2.3	-2.7	-4.9	1.1
255	-1.9	-1.9	-1.9	-4.9	1.1
256	-1.1	-1.1	-1.5	-4.9	1.1
257	-0.3	-0.3	-1.1	-4.9	1.1
258	0.5	0.5	-0.7	-4.9	1.1
259	1.3	1.3	-0.3	-4.9	1.1
260	2.1	2.1	0.1	-4.9	1.1
261	2.9	2.9	0.5	-4.9	1.1
262	3.7	3.7	0.9	-4.9	1.1
263	4.5	4.5	1.3	-4.9	1.1

264	4.5	4.5	1.3	-4.9	1.1
265	3.7	3.7	0.9	-4.9	1.1
266	2.9	2.9	0.5	-4.9	1.1
267	2.1	2.1	0.1	-4.9	1.1
268	1.3	1.3	-0.3	-4.9	1.1
269	0.5	0.5	-0.7	-4.9	1.1
270	-0.3	-0.3	-1.1	-4.9	1.1
271	-1.1	-1.1	-1.5	-4.9	1.1
272	-1.9	-1.9	-1.9	-4.9	1.1
273	-2.7	-2.3	-2.7	-4.9	1.1
274	-3.5	-2.7	-3.5	-4.9	1.1
275	-4.3	-3.1	-4.3	-4.9	1.1
276	-5.1	-3.5	-5.1	-4.9	1.1
277	-5.9	-3.9	-5.9	-4.9	1.1
278	-6.7	-4.3	-6.7	-4.9	1.1
279	-6.7	-4.3	-6.7	-4.9	1.1
280	-6.7	-4.3	-6.7	-4.9	1.1
281	-6.7	-4.3	-6.7	-4.9	1.1
282	-6.7	-4.3	-6.7	-4.9	1.1
283	-6.7	-4.3	-6.7	-4.9	1.1
284	-6.7	-4.3	-6.7	-4.9	1.1
285	-6.7	-4.3	-6.7	-4.9	1.1
286	-6.7	-4.3	-6.7	-4.9	1.1
287	-6.7	-4.3	-6.7	-4.9	1.1
288	-6.7	-4.3	-6.7	-4.9	1.1
289	-6.7	-4.3	-6.7	-4.9	1.1
290	-6.7	-4.3	-6.7	-4.9	1.1
291	-6.7	-4.3	-6.7	-4.9	1.1
292	-6.7	-4.3	-6.7	-4.9	1.1
293	-6.7	-4.3	-6.7	-4.9	1.1
294	-6.7	-4.3	-6.7	-4.9	1.1
295	-6.7	-4.3	-6.7	-4.9	1.1
296	-6.7	-4.3	-6.7	-4.9	1.1
297	-6.7	-4.3	-6.7	-4.9	1.1
298	-6.7	-4.3	-6.7	-4.9	1.1
299	-6.7	-4.3	-6.7	-4.9	1.1
300	-6.7	-4.3	-6.7	-4.9	1.1
301	-6.7	-4.3	-6.7	-4.9	1.1
302	-6.7	-4.3	-6.7	-4.9	1.1
303	-6.7	-4.3	-6.7	-4.9	1.1

304	-6.7	-4.3	-6.7	-4.9	1.1
305	-6.7	-4.3	-6.7	-4.9	1.1
306	-6.7	-4.3	-6.7	-4.9	1.1
307	-6.7	-4.3	-6.7	-4.9	1.1
308	-6.7	-4.3	-6.7	-4.9	1.1
309	-6.7	-4.3	-6.7	-4.9	1.1
310	-6.7	-4.3	-6.7	-4.9	1.1
311	-6.7	-4.3	-6.7	-4.9	1.1
312	-6.7	-4.3	-6.7	-4.9	1.1
313	-6.7	-4.3	-6.7	-4.9	1.1
314	-6.7	-4.3	-6.7	-4.9	1.1
315	-6.7	-4.3	-6.7	-4.9	1.1
316	-6.7	-4.3	-6.7	-4.9	1.1
317	-6.7	-4.3	-6.7	-4.9	1.1
318	-6.7	-4.3	-6.7	-4.9	1.1
319	-6.7	-4.3	-6.7	-4.9	1.1
320	-6.7	-4.3	-6.7	-4.9	1.1
321	-6.7	-4.3	-6.7	-4.9	1.1
322	-6.7	-4.3	-6.7	-4.9	1.1
323	-6.7	-4.3	-6.7	-4.9	1.1
324	-6.7	-4.3	-6.7	-4.9	1.1
325	-6.7	-4.3	-6.7	-4.9	1.1
326	-6.7	-4.3	-6.7	-4.9	1.1
327	-6.7	-4.3	-6.7	-4.9	1.1
328	-6.7	-4.3	-6.7	-4.9	1.1
329	-6.7	-4.3	-6.7	-4.9	1.1
330	-6.7	-4.3	-6.7	-4.9	1.1
331	-6.7	-4.3	-6.7	-4.9	1.1
332	-6.7	-4.3	-6.7	-4.9	1.1
333	-6.7	-4.3	-6.7	-4.9	1.1
334	-6.7	-4.3	-6.7	-4.9	1.1
335	-6.7	-4.3	-6.7	-4.9	1.1
336	-6.7	-4.3	-6.7	-4.9	1.1
337	-6.7	-4.3	-6.7	-4.9	1.1
338	-6.7	-4.3	-6.7	-4.9	1.1
339	-6.7	-4.3	-6.7	-4.9	1.1
340	-5.9	-3.9	-5.9	-4.9	1.1
341	-5.1	-3.5	-5.1	-4.9	1.1
342	-4.3	-3.1	-4.3	-4.9	1.1
343	-3.5	-2.7	-3.5	-4.9	1.1

344	-2.7	-2.3	-2.7	-4.9	1.1
345	-1.9	-1.9	-1.9	-4.9	1.1
346	-1.1	-1.1	-1.5	-4.9	1.1
347	-0.3	-0.3	-1.1	-4.9	1.1
348	0.5	0.5	-0.7	-4.9	1.1
349	1.3	1.3	-0.3	-4.9	1.1
350	2.1	2.1	0.1	-4.9	1.1
351	2.9	2.9	0.5	-4.9	1.1
352	3.7	3.7	0.9	-4.9	1.1
353	4.5	4.5	1.3	-4.9	1.1
354	4.5	4.5	1.3	-4.9	1.1
355	3.7	3.7	0.9	-4.9	1.1
356	2.9	2.9	0.5	-4.9	1.1
357	2.1	2.1	0.1	-4.9	1.1
358	1.3	1.3	-0.3	-4.9	1.1
359	0.5	0.5	-0.7	-4.9	1.1
360	-0.3	-0.3	-1.1	-4.9	1.1
361	-1.1	-1.1	-1.5	-4.9	1.1
362	-1.9	-1.9	-1.9	-4.9	1.1
363	-2.7	-2.3	-2.7	-4.9	1.1
364	-3.5	-2.7	-3.5	-4.9	1.1
365	-4.3	-3.1	-4.3	-4.9	1.1
366	-5.1	-3.5	-5.1	-4.9	1.1
367	-5.9	-3.9	-5.9	-4.9	1.1
368	-6.7	-4.3	-6.7	-4.9	1.1
369	-6.7	-4.3	-6.7	-4.9	1.1
370	-6.7	-4.3	-6.7	-4.9	1.1
371	-6.7	-4.3	-6.7	-4.9	1.1
372	-6.7	-4.3	-6.7	-4.9	1.1
373	-6.7	-4.3	-6.7	-4.9	1.1
374	-6.7	-4.3	-6.7	-4.9	1.1
375	-6.7	-4.3	-6.7	-4.9	1.1
376	-6.7	-4.3	-6.7	-4.9	1.1
377	-6.7	-4.3	-6.7	-4.9	1.1
378	-6.7	-4.3	-6.7	-4.9	1.1
379	-6.7	-4.3	-6.7	-4.9	1.1
380	-6.7	-4.3	-6.7	-4.9	1.1
381	-6.7	-4.3	-6.7	-4.9	1.1
382	-6.7	-4.3	-6.7	-4.9	1.1
383	-6.7	-4.3	-6.7	-4.9	1.1

384	-6.7	-4.3	-6.7	-4.9	1.1
385	-6.7	-4.3	-6.7	-4.9	1.1
386	-6.7	-4.3	-6.7	-4.9	1.1
387	-6.7	-4.3	-6.7	-4.9	1.1
388	-6.7	-4.3	-6.7	-4.9	1.1
389	-6.7	-4.3	-6.7	-4.9	1.1
390	-6.7	-4.3	-6.7	-4.9	1.1
391	-6.7	-4.3	-6.7	-4.9	1.1
392	-6.7	-4.3	-6.7	-4.9	1.1
393	-6.7	-4.3	-6.7	-4.9	1.1
394	-6.7	-4.3	-6.7	-4.9	1.1
395	-6.7	-4.3	-6.7	-4.9	1.1
396	-6.7	-4.3	-6.7	-4.9	1.1
397	-6.7	-4.3	-6.7	-4.9	1.1
398	-6.7	-4.3	-6.7	-4.9	1.1
399	-6.7	-4.3	-6.7	-4.9	1.1
400	-6.7	-4.3	-6.7	-4.9	1.1
401	-6.7	-4.3	-6.7	-4.9	1.1
402	-6.7	-4.3	-6.7	-4.9	1.1
403	-6.7	-4.3	-6.7	-4.9	1.1
404	-6.7	-4.3	-6.7	-4.9	1.1
405	-6.7	-4.3	-6.7	-4.9	1.1
406	-6.7	-4.3	-6.7	-4.9	1.1
407	-6.7	-4.3	-6.7	-4.9	1.1
408	-6.7	-4.3	-6.7	-4.9	1.1
409	-6.7	-4.3	-6.7	-4.9	1.1
410	-6.7	-4.3	-6.7	-4.9	1.1
411	-6.7	-4.3	-6.7	-4.9	1.1
412	-6.7	-4.3	-6.7	-4.9	1.1
413	-6.7	-4.3	-6.7	-4.9	1.1
414	-6.7	-4.3	-6.7	-4.9	1.1
415	-6.7	-4.3	-6.7	-4.9	1.1
416	-6.7	-4.3	-6.7	-4.9	1.1
417	-6.7	-4.3	-6.7	-4.9	1.1
418	-6.7	-4.3	-6.7	-4.9	1.1
419	-6.7	-4.3	-6.7	-4.9	1.1
420	-6.7	-4.3	-6.7	-4.9	1.1
421	-6.7	-4.3	-6.7	-4.9	1.1
422	-6.7	-4.3	-6.7	-4.9	1.1
423	-6.7	-4.3	-6.7	-4.9	1.1

424	-6.7	-4.3	-6.7	-4.9	1.1
425	-6.7	-4.3	-6.7	-4.9	1.1
426	-6.7	-4.3	-6.7	-4.9	1.1
427	-6.7	-4.3	-6.7	-4.9	1.1
428	-6.7	-4.3	-6.7	-4.9	1.1
429	-5.9	-3.9	-5.9	-4.9	1.1
430	-5.1	-3.5	-5.1	-4.9	1.1
431	-4.3	-3.1	-4.3	-4.9	1.1
432	-3.5	-2.7	-3.5	-4.9	1.1
433	-2.7	-2.3	-2.7	-4.9	1.1
434	-1.9	-1.9	-1.9	-4.9	1.1
435	-1.1	-1.1	-1.5	-4.9	1.1
436	-0.3	-0.3	-1.1	-4.9	1.1
437	0.5	0.5	-0.7	-4.9	1.1
438	1.3	1.3	-0.3	-4.9	1.1
439	2.1	2.1	0.1	-4.9	1.1
440	2.9	2.9	0.5	-4.9	1.1
441	3.7	3.7	0.9	-4.9	1.1
442	4.5	4.5	1.3	-4.9	1.1
443	4.5	4.5	1.3	-4.9	1.1
444	3.7	3.7	0.9	-4.9	1.1
445	2.9	2.9	0.5	-4.9	1.1
446	2.1	2.1	0.1	-4.9	1.1
447	1.3	1.3	-0.3	-4.9	1.1
448	0.5	0.5	-0.7	-4.9	1.1
449	-0.3	-0.3	-1.1	-4.9	1.1
450	-1.1	-1.1	-1.5	-4.9	1.1
451	-1.9	-1.9	-1.9	-4.9	1.1
452	-2.7	-2.3	-2.7	-4.9	1.1
453	-3.5	-2.7	-3.5	-4.9	1.1
454	-4.3	-3.1	-4.3	-4.9	1.1
455	-5.1	-3.5	-5.1	-4.9	1.1
456	-5.9	-3.9	-5.9	-4.9	1.1
457	-6.7	-4.3	-6.7	-4.9	1.1
458	-6.7	-4.3	-6.7	-4.9	1.1
459	-6.7	-4.3	-6.7	-4.9	1.1
460	-6.7	-4.3	-6.7	-4.9	1.1
461	-6.7	-4.3	-6.7	-4.9	1.1
462	-6.7	-4.3	-6.7	-4.9	1.1
463	-6.7	-4.3	-6.7	-4.9	1.1

464	-6.7	-4.3	-6.7	-4.9	1.1
465	-6.7	-4.3	-6.7	-4.9	1.1
466	-6.7	-4.3	-6.7	-4.9	1.1
467	-6.7	-4.3	-6.7	-4.9	1.1
468	-6.7	-4.3	-6.7	-4.9	1.1
469	-6.7	-4.3	-6.7	-4.9	1.1
470	-6.7	-4.3	-6.7	-4.9	1.1
471	-6.7	-4.3	-6.7	-4.9	1.1
472	-6.7	-4.3	-6.7	-4.9	1.1
473	-6.7	-4.3	-6.7	-4.9	1.1
474	-6.7	-4.3	-6.7	-4.9	1.1
475	-6.7	-4.3	-6.7	-4.9	1.1
476	-6.7	-4.3	-6.7	-4.9	1.1
477	-6.7	-4.3	-6.7	-4.9	1.1
478	-6.7	-4.3	-6.7	-4.9	1.1
479	-6.7	-4.3	-6.7	-4.9	1.1
480	-6.7	-4.3	-6.7	-4.9	1.1
481	-6.7	-4.3	-6.7	-4.9	1.1
482	-6.7	-4.3	-6.7	-4.9	1.1
483	-6.7	-4.3	-6.7	-4.9	1.1
484	-6.7	-4.3	-6.7	-4.9	1.1
485	-6.7	-4.3	-6.7	-4.9	1.1
486	-6.7	-4.3	-6.7	-4.9	1.1
487	-6.7	-4.3	-6.7	-4.9	1.1
488	-6.7	-4.3	-6.7	-4.9	1.1
489	-6.7	-4.3	-6.7	-4.9	1.1
490	-6.7	-4.3	-6.7	-4.9	1.1
491	-6.7	-4.3	-6.7	-4.9	1.1
492	-6.7	-4.3	-6.7	-4.9	1.1
493	-6.7	-4.3	-6.7	-4.9	1.1
494	-6.7	-4.3	-6.7	-4.9	1.1
495	-6.7	-4.3	-6.7	-4.9	1.1
496	-6.7	-4.3	-6.7	-4.9	1.1
497	-6.7	-4.3	-6.7	-4.9	1.1
498	-6.7	-4.3	-6.7	-4.9	1.1
499	-6.7	-4.3	-6.7	-4.9	1.1
500	-6.7	-4.3	-6.7	-4.9	1.1
501	-6.7	-4.3	-6.7	-4.9	1.1
502	-6.7	-4.3	-6.7	-4.9	1.1
503	-6.7	-4.3	-6.7	-4.9	1.1

504	-6.7	-4.3	-6.7	-4.9	1.1
505	-6.7	-4.3	-6.7	-4.9	1.1
506	-6.7	-4.3	-6.7	-4.9	1.1
507	-6.7	-4.3	-6.7	-4.9	1.1
508	-6.7	-4.3	-6.7	-4.9	1.1
509	-6.7	-4.3	-6.7	-4.9	1.1
510	-6.7	-4.3	-6.7	-4.9	1.1
511	-6.7	-4.3	-6.7	-4.9	1.1
512	-6.7	-4.3	-6.7	-4.9	1.1
513	-6.7	-4.3	-6.7	-4.9	1.1
514	-6.7	-4.3	-6.7	-4.9	1.1
515	-6.7	-4.3	-6.7	-4.9	1.1
516	-6.7	-4.3	-6.7	-4.9	1.1
517	-6.7	-4.3	-6.7	-4.9	1.1
518	-6.7	-4.3	-6.7	-4.9	1.1
519	-5.9	-3.9	-5.9	-4.9	1.1
520	-5.1	-3.5	-5.1	-4.9	1.1
521	-4.3	-3.1	-4.3	-4.9	1.1
522	-3.5	-2.7	-3.5	-4.9	1.1
523	-2.7	-2.3	-2.7	-4.9	1.1
524	-1.9	-1.9	-1.9	-4.9	1.1
525	-1.1	-1.1	-1.5	-4.9	1.1
526	-0.3	-0.3	-1.1	-4.9	1.1
527	0.5	0.5	-0.7	-4.9	1.1
528	1.3	1.3	-0.3	-4.9	1.1
529	2.1	2.1	0.1	-4.9	1.1
530	2.9	2.9	0.5	-4.9	1.1
531	3.7	3.7	0.9	-4.9	1.1
532	4.5	4.5	1.3	-4.9	1.1
533	4.5	4.5	1.3	-4.9	1.1
534	3.7	3.7	0.9	-4.9	1.1
535	2.9	2.9	0.5	-4.9	1.1
536	2.1	2.1	0.1	-4.9	1.1
537	1.3	1.3	-0.3	-4.9	1.1
538	0.5	0.5	-0.7	-4.9	1.1
539	-0.3	-0.3	-1.1	-4.9	1.1
540	-1.1	-1.1	-1.5	-4.9	1.1
541	-1.9	-1.9	-1.9	-4.9	1.1
542	-2.7	-2.3	-2.7	-4.9	1.1
543	-3.5	-2.7	-3.5	-4.9	1.1

544	-4.3	-3.1	-4.3	-4.9	1.1
545	-5.1	-3.5	-5.1	-4.9	1.1
546	-5.9	-3.9	-5.9	-4.9	1.1
547	-6.7	-4.3	-6.7	-4.9	1.1
548	-6.7	-4.3	-6.7	-4.9	1.1
549	-6.7	-4.3	-6.7	-4.9	1.1
550	-6.7	-4.3	-6.7	-4.9	1.1
551	-6.7	-4.3	-6.7	-4.9	1.1
552	-6.7	-4.3	-6.7	-4.9	1.1
553	-6.7	-4.3	-6.7	-4.9	1.1
554	-6.7	-4.3	-6.7	-4.9	1.1
555	-6.7	-4.3	-6.7	-4.9	1.1
556	-6.7	-4.3	-6.7	-4.9	1.1
557	-6.7	-4.3	-6.7	-4.9	1.1
558	-6.7	-4.3	-6.7	-4.9	1.1
559	-6.7	-4.3	-6.7	-4.9	1.1
560	-6.7	-4.3	-6.7	-4.9	1.1
561	-6.7	-4.3	-6.7	-4.9	1.1
562	-6.7	-4.3	-6.7	-4.9	1.1
563	-6.7	-4.3	-6.7	-4.9	1.1
564	-6.7	-4.3	-6.7	-4.9	1.1
565	-6.7	-4.3	-6.7	-4.9	1.1
566	-6.7	-4.3	-6.7	-4.9	1.1
567	-6.7	-4.3	-6.7	-4.9	1.1
568	-6.7	-4.3	-6.7	-4.9	1.1
569	-6.7	-4.3	-6.7	-4.9	1.1
570	-6.7	-4.3	-6.7	-4.9	1.1
571	-6.7	-4.3	-6.7	-4.9	1.1
572	-6.7	-4.3	-6.7	-4.9	1.1
573	-6.7	-4.3	-6.7	-4.9	1.1
574	-6.7	-4.3	-6.7	-4.9	1.1
575	-6.7	-4.3	-6.7	-4.9	1.1
576	-6.7	-4.3	-6.7	-4.9	1.1
577	-6.7	-4.3	-6.7	-4.9	1.1
578	-6.7	-4.3	-6.7	-4.9	1.1
579	-6.7	-4.3	-6.7	-4.9	1.1
580	-6.7	-4.3	-6.7	-4.9	1.1
581	-6.7	-4.3	-6.7	-4.9	1.1
582	-6.7	-4.3	-6.7	-4.9	1.1
583	-6.7	-4.3	-6.7	-4.9	1.1

584	-6.7	-4.3	-6.7	-4.9	1.1
585	-6.7	-4.3	-6.7	-4.9	1.1
586	-6.7	-4.3	-6.7	-4.9	1.1
587	-6.7	-4.3	-6.7	-4.9	1.1
588	-6.7	-4.3	-6.7	-4.9	1.1
589	-6.7	-4.3	-6.7	-4.9	1.1
590	-6.7	-4.3	-6.7	-4.9	1.1
591	-6.7	-4.3	-6.7	-4.9	1.1
592	-6.7	-4.3	-6.7	-4.9	1.1
593	-6.7	-4.3	-6.7	-4.9	1.1
594	-6.7	-4.3	-6.7	-4.9	1.1
595	-6.7	-4.3	-6.7	-4.9	1.1
596	-6.7	-4.3	-6.7	-4.9	1.1
597	-6.7	-4.3	-6.7	-4.9	1.1
598	-6.7	-4.3	-6.7	-4.9	1.1
599	-6.7	-4.3	-6.7	-4.9	1.1
600	-6.7	-4.3	-6.7	-4.9	1.1
601	-6.7	-4.3	-6.7	-4.9	1.1
602	-6.7	-4.3	-6.7	-4.9	1.1
603	-6.7	-4.3	-6.7	-4.9	1.1
604	-6.7	-4.3	-6.7	-4.9	1.1
605	-6.7	-4.3	-6.7	-4.9	1.1
606	-6.7	-4.3	-6.7	-4.9	1.1
607	-6.7	-4.3	-6.7	-4.9	1.1
608	-5.9	-3.9	-5.9	-4.9	1.1
609	-5.1	-3.5	-5.1	-4.9	1.1
610	-4.3	-3.1	-4.3	-4.9	1.1
611	-3.5	-2.7	-3.5	-4.9	1.1
612	-2.7	-2.3	-2.7	-4.9	1.1
613	-1.9	-1.9	-1.9	-4.9	1.1
614	-1.1	-1.1	-1.5	-4.9	1.1
615	-0.3	-0.3	-1.1	-4.9	1.1
616	0.5	0.5	-0.7	-4.9	1.1
617	1.3	1.3	-0.3	-4.9	1.1
618	2.1	2.1	0.1	-4.9	1.1
619	2.9	2.9	0.5	-4.9	1.1
620	3.7	3.7	0.9	-4.9	1.1
621	4.5	4.5	1.3	-4.9	1.1
622	4.5	4.5	1.3	-4.9	1.1
623	3.7	3.7	0.9	-4.9	1.1

624	2.9	2.9	0.5	-4.9	1.1
625	2.1	2.1	0.1	-4.9	1.1
626	1.3	1.3	-0.3	-4.9	1.1
627	0.5	0.5	-0.7	-4.9	1.1
628	-0.3	-0.3	-1.1	-4.9	1.1
629	-1.1	-1.1	-1.5	-4.9	1.1
630	-1.9	-1.9	-1.9	-4.9	1.1
631	-2.7	-2.3	-2.7	-4.9	1.1
632	-3.5	-2.7	-3.5	-4.9	1.1
633	-4.3	-3.1	-4.3	-4.9	1.1
634	-5.1	-3.5	-5.1	-4.9	1.1
635	-5.9	-3.9	-5.9	-4.9	1.1
636	-6.7	-4.3	-6.7	-4.9	1.1
637	-6.7	-4.3	-6.7	-4.9	1.1
638	-6.7	-4.3	-6.7	-4.9	1.1
639	-6.7	-4.3	-6.7	-4.9	1.1
640	-6.7	-4.3	-6.7	-4.9	1.1
641	-6.7	-4.3	-6.7	-4.9	1.1
642	-6.7	-4.3	-6.7	-4.9	1.1
643	-6.7	-4.3	-6.7	-4.9	1.1
644	-6.7	-4.3	-6.7	-4.9	1.1
645	-6.7	-4.3	-6.7	-4.9	1.1
646	-6.7	-4.3	-6.7	-4.9	1.1
647	-6.7	-4.3	-6.7	-4.9	1.1
648	-6.7	-4.3	-6.7	-4.9	1.1
649	-6.7	-4.3	-6.7	-4.9	1.1
650	-6.7	-4.3	-6.7	-4.9	1.1
651	-6.7	-4.3	-6.7	-4.9	1.1
652	-6.7	-4.3	-6.7	-4.9	1.1
653	-6.7	-4.3	-6.7	-4.9	1.1
654	-6.7	-4.3	-6.7	-4.9	1.1
655	-6.7	-4.3	-6.7	-4.9	1.1
656	-6.7	-4.3	-6.7	-4.9	1.1
657	-6.7	-4.3	-6.7	-4.9	1.1
658	-6.7	-4.3	-6.7	-4.9	1.1
659	-6.7	-4.3	-6.7	-4.9	1.1
660	-6.7	-4.3	-6.7	-4.9	1.1
661	-6.7	-4.3	-6.7	-4.9	1.1
662	-6.7	-4.3	-6.7	-4.9	1.1
663	-6.7	-4.3	-6.7	-4.9	1.1

664	-6.7	-4.3	-6.7	-4.9	1.1
665	-6.7	-4.3	-6.7	-4.9	1.1
666	-6.7	-4.3	-6.7	-4.9	1.1
667	-6.7	-4.3	-6.7	-4.9	1.1
668	-6.7	-4.3	-6.7	-4.9	1.1
669	-6.7	-4.3	-6.7	-4.9	1.1
670	-6.7	-4.3	-6.7	-4.9	1.1
671	-6.7	-4.3	-6.7	-4.9	1.1
672	-6.7	-4.3	-6.7	-4.9	1.1
673	-6.7	-4.3	-6.7	-4.9	1.1
674	-6.7	-4.3	-6.7	-4.9	1.1
675	-6.7	-4.3	-6.7	-4.9	1.1
676	-6.7	-4.3	-6.7	-4.9	1.1
677	-6.7	-4.3	-6.7	-4.9	1.1
678	-6.7	-4.3	-6.7	-4.9	1.1
679	-6.7	-4.3	-6.7	-4.9	1.1
680	-6.7	-4.3	-6.7	-4.9	1.1
681	-6.7	-4.3	-6.7	-4.9	1.1
682	-6.7	-4.3	-6.7	-4.9	1.1
683	-6.7	-4.3	-6.7	-4.9	1.1
684	-6.7	-4.3	-6.7	-4.9	1.1
685	-6.7	-4.3	-6.7	-4.9	1.1
686	-6.7	-4.3	-6.7	-4.9	1.1
687	-6.7	-4.3	-6.7	-4.9	1.1
688	-6.7	-4.3	-6.7	-4.9	1.1
689	-6.7	-4.3	-6.7	-4.9	1.1
690	-6.7	-4.3	-6.7	-4.9	1.1
691	-6.7	-4.3	-6.7	-4.9	1.1
692	-6.7	-4.3	-6.7	-4.9	1.1
693	-6.7	-4.3	-6.7	-4.9	1.1
694	-6.7	-4.3	-6.7	-4.9	1.1
695	-6.7	-4.3	-6.7	-4.9	1.1
696	-6.7	-4.3	-6.7	-4.9	1.1
697	-6.7	-4.3	-6.7	-4.9	1.1
698	-6.7	-4.3	-6.7	-4.9	1.1
699	-6.7	-4.3	-6.7	-4.9	1.1
700	-6.7	-4.3	-6.7	-4.9	1.1
701	-5.9	-3.9	-5.9	-4.9	1.1
702	-5.1	-3.5	-5.1	-4.9	1.1
703	-4.3	-3.1	-4.3	-4.9	1.1

704	-3.5	-2.7	-3.5	-4.9	1.1
705	-2.7	-2.3	-2.7	-4.9	1.1
706	-1.9	-1.9	-1.9	-4.9	1.1
707	-1.1	-1.1	-1.5	-4.9	1.1
708	-0.3	-0.3	-1.1	-4.9	1.1
709	0.5	0.5	-0.7	-4.9	1.1
710	1.3	1.3	-0.3	-4.9	1.1
711	2.1	2.1	0.1	-4.9	1.1
712	2.9	2.9	0.5	-4.9	1.1
713	3.7	3.7	0.9	-4.9	1.1
714	4.5	4.5	1.3	-4.9	1.1
715	4.5	4.5	1.3	-4.9	1.1
716	3.7	3.7	0.9	-4.9	1.1
717	2.9	2.9	0.5	-4.9	1.1
718	2.1	2.1	0.1	-4.9	1.1
719	1.3	1.3	-0.3	-4.9	1.1
720	1.3	0.5	-0.7	-4.9	1.1

9
