# Supplementary material for: Experimental evidence demonstrating how freeze-thaw patterns affect spoilage of perishable cached food
Source: PLoS One. 2025 Apr 4;20(4):e0319043. doi: 10.1371/journal.pone.0319043 (PMC11970643; doi:10.1371/journal.pone.0319043)
Supplement: S6 Table — Caches consisted of 1.20g of raw chicken breast placed between two pieces of black spruce (Picea mariana) bark. (PDF) [file pone.0319043.s006.pdf]

S6 Table. Weight loss of caches in experiment 1 that tested the predictions of the '*exacerbation hypothesis*'. Caches consisted of 1.20g of raw chicken breast placed between two pieces of black spruce (*Picea mariana*) bark.

Treatment	Sample ID	Starting weight (g)	End weight (g)	Proportional weight loss
High temperature control	CA1	1.2	0.37	0.69
High temperature control	CA2	1.2	0.38	0.68
High temperature control	CA3	1.2	0.38	0.68
High temperature control	CA4	1.2	0.38	0.68
High temperature control	CA5	1.2	0.41	0.66
High temperature control	CA6	1.2	0.4	0.67
High temperature control	CA7	1.2	0.41	0.66
High temperature control	CA8	1.2	0.41	0.66
High temperature control	CA9	1.2	0.41	0.66
Low temperature control	CB1	1.2	0.41	0.66
Low temperature control	CB2	1.2	0.41	0.66
Low temperature control	CB3	1.2	0.41	0.66
Low temperature control	CB4	1.2	0.42	0.65
Low temperature control	CB5	1.2	0.42	0.65
Low temperature control	CB6	1.2	0.42	0.65
Low temperature control	CB7	1.2	0.41	0.66
Low temperature control	CB8	1.2	0.42	0.65
Low temperature control	CB9	1.2	0.42	0.65
Early freeze-thaw	E1	1.2	0.35	0.71
Early freeze-thaw	E2	1.2	0.35	0.71
Early freeze-thaw	E3	1.2	0.34	0.72
Early freeze-thaw	E4	1.2	0.33	0.72
Early freeze-thaw	E5	1.2	0.34	0.71
Early freeze-thaw	E6	1.2	0.34	0.71
Early freeze-thaw	E7	1.2	0.34	0.71
Early freeze-thaw	E8	1.2	0.34	0.72
Early freeze-thaw	E9	1.2	0.35	0.71
Late freeze-thaw	L1	1.2	0.34	0.71
Late freeze-thaw	L2	1.2	0.35	0.71
Late freeze-thaw	L3	1.2	0.42	0.65
Late freeze-thaw	L4	1.2	0.43	0.64

Late freeze-thaw	L5	1.2	0.43	0.64
Late freeze-thaw	L6	1.2	0.44	0.63
Late freeze-thaw	L7	1.2	0.43	0.64
Late freeze-thaw	L8	1.2	0.42	0.65
Late freeze-thaw	L9	1.2	0.42	0.65
