# Supplementary material for: Experimental evidence demonstrating how freeze-thaw patterns affect spoilage of perishable cached food
Source: PLoS One. 2025 Apr 4;20(4):e0319043. doi: 10.1371/journal.pone.0319043 (PMC11970643; doi:10.1371/journal.pone.0319043)
Supplement: S7 Table — Caches consisted of 1.20g of raw chicken breast placed between two pieces of black spruce (Picea mariana) bark. (PDF) [file pone.0319043.s007.pdf]

S7 Table. Weight loss of caches in experiment 2 that tested the predictions of the '*frequency hypothesis'* and the '*continuous thaw hypothesis*'. Caches consisted of 1.20g of raw chicken breast placed between two pieces of black spruce (*Picea mariana*) bark.

Treatment	Sample ID	Starting weight (g)	final weight (g)	Proportional weight loss
Low freq. freeze-thaw	L1	1.2	0.32	0.73
Low freq. freeze-thaw	L2	1.2	0.33	0.73
Low freq. freeze-thaw	L3	1.2	0.31	0.74
Low freq. freeze-thaw	L4	1.2	0.32	0.73
Low freq. freeze-thaw	L5	1.2	0.32	0.73
Low freq. freeze-thaw	L6	1.2	0.34	0.72
Low freq. freeze-thaw	L7	1.2	0.32	0.73
Low freq. freeze-thaw	L8	1.2	0.34	0.72
Low freq. freeze-thaw	L9	1.2	0.33	0.73
Med freq. freeze-thaw	M1	1.2	0.36	0.70
Med freq. freeze-thaw	M2	1.2	0.35	0.71
Med freq. freeze-thaw	M3	1.2	0.36	0.70
Med freq. freeze-thaw	M4	1.2	0.38	0.68
Med freq. freeze-thaw	M5	1.2	0.34	0.72
Med freq. freeze-thaw	M6	1.2	0.36	0.70
Med freq. freeze-thaw	M7	1.2	0.35	0.71
Med freq. freeze-thaw	M8	1.2	0.35	0.71
Med freq. freeze-thaw	M9	1.2	0.35	0.71
High freq. freeze-thaw	H1	1.2	0.35	0.71
High freq. freeze-thaw	H2	1.2	0.37	0.69
High freq. freeze-thaw	H3	1.2	0.37	0.69
High freq. freeze-thaw	H4	1.2	0.37	0.69
High freq. freeze-thaw	H5	1.2	0.35	0.71
High freq. freeze-thaw	H6	1.2	0.39	0.68
High freq. freeze-thaw	H7	1.2	0.36	0.70
High freq. freeze-thaw	H8	1.2	0.36	0.70
High freq. freeze-thaw	H9	1.2	0.37	0.69
Control	C1	1.2	0.33	0.73
Control	C2	1.2	0.32	0.73
Control	C3	1.2	0.32	0.73
Control	C4	1.2	0.33	0.73
Control	C5	1.2	0.31	0.74
Control	C6	1.2	0.33	0.73

Control	C7	1.2	0.33	0.73
Control	C8	1.2	0.32	0.73
Control	C9	1.2	0.33	0.73
