# Supplementary material for: Experimental evidence demonstrating how freeze-thaw patterns affect spoilage of perishable cached food
Source: PLoS One. 2025 Apr 4;20(4):e0319043. doi: 10.1371/journal.pone.0319043 (PMC11970643; doi:10.1371/journal.pone.0319043)
Supplement: S8 Table — Caches consisted of 1.20g of raw chicken breast placed between two pieces of black spruce (Picea mariana) bark. (PDF) [file pone.0319043.s008.pdf]

S8 Table. Weight loss of caches in experiment 3 that tested the predictions of the '*freeze intensity hypothesis*' and '*thaw intensity hypothesis*'. Caches consisted of 1.20g of raw chicken breast placed between two pieces of black spruce (*Picea mariana*) bark.

Treatment	Sample ID	Starting weight (g)	End weight (g)	Proportional weight loss
Average freeze-thaw	HT1	1.20	0.37	0.69
Average freeze-thaw	HT2	1.20	0.39	0.68
Average freeze-thaw	HT3	1.20	0.38	0.68
Average freeze-thaw	HT4	1.20	0.38	0.69
Average freeze-thaw	HT5	1.20	0.39	0.68
Average freeze-thaw	HT6	1.20	0.38	0.69
Average freeze-thaw	HT7	1.20	0.38	0.69
Average freeze-thaw	HT8	1.20	0.38	0.69
Average freeze-thaw	HT9	1.20	0.37	0.69
Mild thaw	WT1	1.20	0.35	0.71
Mild thaw	WT2	1.20	0.34	0.72
Mild thaw	WT3	1.20	0.34	0.72
Mild thaw	WT4	1.20	0.34	0.72
Mild thaw	WT5	1.20	0.34	0.72
Mild thaw	WT6	1.20	0.33	0.73
Mild thaw	WT7	1.20	0.35	0.71
Mild thaw	WT8	1.20	0.35	0.71
Mild thaw	WT9	1.20	0.33	0.73
Mild freeze	WF1	1.20	0.35	0.71
Mild freeze	WF2	1.20	0.35	0.71
Mild freeze	WF3	1.20	0.34	0.72
Mild freeze	WF4	1.20	0.34	0.72
Mild freeze	WF5	1.20	0.34	0.72
Mild freeze	WF6	1.20	0.34	0.72
Mild freeze	WF7	1.20	0.35	0.71
Mild freeze	WF8	1.20	0.34	0.72
Mild freeze	WF9	1.20	0.34	0.72
Low temperature control	LC1	1.20	0.33	0.73
Low temperature control	LC2	1.20	0.33	0.73
Low temperature control	LC3	1.20	0.34	0.72
Low temperature control	LC4	1.20	0.34	0.72
Low temperature control	LC5	1.20	0.34	0.72
Low temperature control	LC6	1.20	0.34	0.72

Low temperature control	LC7	1.20	0.34	0.72
Low temperature control	LC8	1.20	0.33	0.73
Low temperature control	LC9	1.20	0.34	0.72
High temperature control	HC1	1.20	0.31	0.75
High temperature control	HC2	1.20	0.31	0.74
High temperature control	HC3	1.20	0.31	0.75
High temperature control	HC4	1.20	0.30	0.75
High temperature control	HC5	1.20	0.31	0.74
High temperature control	HC6	1.20	0.31	0.74
High temperature control	HC7	1.20	0.30	0.75
High temperature control	HC8	1.20	0.31	0.75
High temperature control	HC9	1.20	0.31	0.74

4
