# Supplementary figures and images for: Experimental evidence demonstrating how freeze-thaw patterns affect spoilage of perishable cached food
Source: PLoS One. 2025 Apr 4;20(4):e0319043. doi: 10.1371/journal.pone.0319043 (PMC11970643; doi:10.1371/journal.pone.0319043)

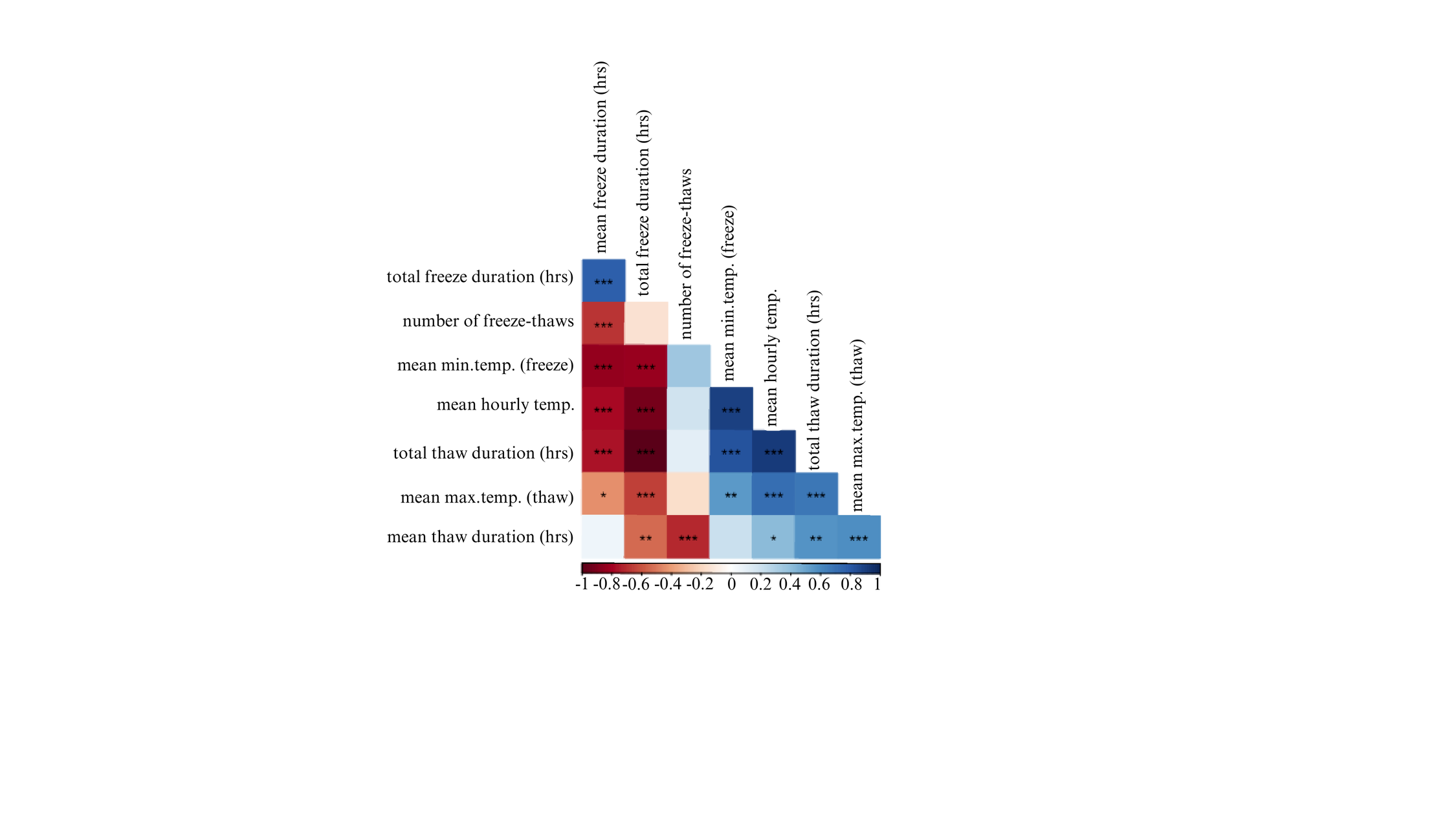

Supplement: S1 Fig — * indicates p < 0.05, ** indicates p < 0.01, and *** indicates p < 0.001. ‘Total freeze duration (hrs)’ is total hours below freezing point for a given year, ‘number of freeze-thaws’ is total number of freeze-thaw events that occurred in Nov. for a given year, ‘mean min. temp. (freeze)’ is mean of the lowest temperatures reached across individual freeze phases for a given year, ‘mean hourly temp.’ is mean temperature of the 720 hrs for each year, ‘total thaw duration (hrs)’ is total hours above freezing point for a given year following the first freeze, ‘mean max. temp. (thaw)’ is mean of the maximum temperatures reach across individual thaw phases for a given year, ‘mean thaw duration (hrs)’ is mean duration in hours for an individual thaw for a given year, and ‘mean freeze duration (hrs)’ is mean duration in hours of individual freeze phases for a given year. Calculations were based on hourly climate data for the month of Nov. from Algonquin Provincial Park East Gate (45°32’N, 78°54’W; 2004 − 2019). (TIF) [file pone.0319043.s009.tif]
