# Supplementary material for: Experimental evidence demonstrating how freeze-thaw patterns affect spoilage of perishable cached food
Source: PLoS One. 2025 Apr 4;20(4):e0319043. doi: 10.1371/journal.pone.0319043 (PMC11970643; doi:10.1371/journal.pone.0319043)
Supplement: Attachment — Submitted filename: Ong_Norris_Response_Letter.docx [file pone.0319043.s012.docx]

Thank you for these comments. Please see below for our responses (in bold text) to each of the comments provided by the two reviewers. We think the manuscript has been improved considerably.

Thank you,

Karen Ong, Ryan Norris

Reviewer #1:

1. Abstract has to be written in a manner that encompass why, how and what obtained from the current research.

**Author response: We have extensively revised the abstract to make sure all of these elements are properly communicated. Specifically, we have added more information on the rationale for the experiment, as well as additional methodological details.**

2. Methods has to be written clearly to be replicable

**Author response: We have also made extensive revisions the methods to improve clarity. We have also added tables in the supplementary material that show hourly programming schedules for all experiments.**

3. The findings have to be presented up to the standard.

**Author response: Our apologies. We are not sure what the reviewer means here. We have edited the results to improve clarity, but the presentation of the results includes all necessarily statistical moments and output from the models.**

4. Discussion is good but need improvement.

**Author response: Given the reviewer has not been specific here, we have carefully gone through the discussion to improve clarity in several places. As a result, we think the discussion has improved considerably.**

5. Conclusion must be based on the findings of the current study.

**Author response: All our conclusions are based on the results of the experiments presented in this paper.**

Reviewer #2:

1. The title is ok and related to the manuscript.

**Author response: Thank you.**

2. Abstract is quite good. Maybe can include some method.

**Author response: Thank you. As suggested, we have included additional information in the abstract about the methods used in our experiments.**

3. Introduction is ok.

**Author response: Thank you.**

4. Methods are well explained.

**Author response: Thank you.**

5. Results are ok. The graphs and diagrams can be view publicly.

**Author response: Thank you.**

6. The discussion is quite good.

**Author response: Thank you. We appreciate the positive comments.**
