# Supplementary material for: Experimental evidence demonstrating how freeze-thaw patterns affect spoilage of perishable cached food
Source: PLoS One. 2025 Apr 4;20(4):e0319043. doi: 10.1371/journal.pone.0319043 (PMC11970643; doi:10.1371/journal.pone.0319043)
Supplement: Attachment — Submitted filename: Reviewer comment.docx [file pone.0319043.s010.docx]

General comments to authors

1. Abstract has to be written in a manner that encompass why, how and what obtained from the current research.

2. Methods has to be written clearly to be replicable

3. The findings have to be presented up to the standard.

4. Discussion is good but need improvement.

5. Conclusion must be based on the findings of the current study.
